# Discriminative ‘Turn-on’ Detection of Al^3+^ and Ga^3+^ Ions as Well as Aspartic Acid by Two Fluorescent Chemosensors

**DOI:** 10.3390/s23041798

**Published:** 2023-02-06

**Authors:** Hina Goyal, Ibrahim Annan, Deepali Ahluwalia, Arijit Bag, Rajeev Gupta

**Affiliations:** 1 Department of Chemistry, University of Delhi, Delhi 110007, India; 2 Department of Applied Chemistry, Maulana Abul Kalam Azad University of Technology, Nadia 742149, India

**Keywords:** Schiff base, sensing, aluminum, AIEE, aspartic acid

## Abstract

In this work, two Schiff-base-based chemosensors **L1** and **L2** containing electron-rich quinoline and anthracene rings were designed. **L1** is AIEE active in a MeOH-H_2_O solvent system while formed aggregates as confirmed by the DLS measurements and fluorescence lifetime studies. The chemosensor **L1** was used for the sensitive, selective, and reversible ‘turn-on’ detection of Al^3+^ and Ga^3+^ ions as well as Aspartic Acid (Asp). Chemosensor **L2**, an isomer of **L1**, was able to selectively detect Ga^3+^ ion even in the presence of Al^3+^ ions and thus was able to discriminate between the two ions. The binding mode of chemosensors with analytes was substantiated through a combination of ^1^H NMR spectra, mass spectra, and DFT studies. The ‘turn-on’ nature of fluorescence sensing by the two chemosensors enabled the development of colorimetric detection, filter-paper-based test strips, and polystyrene film-based detection techniques.

## 1. Introduction

Earth’s crust consists of various metals, out of which aluminum is the third most abundant [1,2]. Aluminum metal and its compounds enter into the environment through various anthropogenic activities such as building materials, transportation, electronics, industrial effluents, wastewater treatment plants, utensils, and food additives [3,4,5,6]. As a result, aluminum gets absorbed and accumulated in the human body due to its excessive exposure [6,7,8]. Although aluminum is a nonessential metal in the human body, once inside, it competes with other important metals with similar properties (e.g., Mg^2+^, Ca^2+^, and Fe^3+^ ions) to bind to various proteins [9,10,11,12]. The high concentration of aluminum in humans can cause severe neurodegenerative diseases such as Alzheimer’s, amyotrophic lateral sclerosis, and Parkinson’s diseases along with various other diseases including microcytic hypochromic anemia, dialysis encephalopathy, and Al-related bone disease [1,13,14,15]. As per the World Health Organization (WHO), the permissible limit of aluminum ions in drinking water is 7.4 μM while the daily average intake should not exceed 3–10 mg [15,16,17]. Therefore, efforts have been made to develop efficient sensors that can detect aluminum ion (Al^3+^) selectively and sensitively [10]. Similarly, gallium ions (Ga^3+^), belonging to the identical IIIA group as Al^3+^, have also been recognized as a potential risk to human health [4,18,19,20].

Amino acids, as building blocks, are essential for living organisms for various vital functions [21,22,23]. Amongst all nonessential amino acids, Aspartic Acid (Asp) plays several critical roles in various biological and physiological processes [24,25,26]. Asp is specifically required for its role in the tricarboxylic acid (TCA) cycle [27,28]. Asp also serves as the main precursor for the synthesis of two amino acids, i.e., Glutamic acid and Glycine [24,29]. Asp acts as a major excitatory neurotransmitter in the mammalian central nervous system [24,30]. Studies have shown the use of K^+^Asp as a cure for heart and liver diseases and diabetes, but a higher amount of Asp can also cause Lou Gehrig’s disease, and it is also connected with the early onset of Alzheimer’s disease [25,27,30]. Therefore, the selective and sensitive detection of Asp is quite essential. The literature presents a number of chemosensors that can detect both Al^3+^ and Ga^3+^ ions, but only a few chemosensors are known for the discriminative detection of Al^3+^ and Ga^3+^ ions [31]. Similarly, a very limited number of examples are available where a chemosensor can detect Al^3+^ and Ga^3+^ ions as well as Asp [31].

Many chemosensors suffer from the Aggregation Caused Quenching (ACQ) at higher concentrations [32,33]. Hence, anti-ACQ materials, also known as the Aggregation-Induced Enhanced Emission (AIEE) materials have generated a lot of interest due to their unique light-emitting properties [34,35]. Such AIEE-active fluorophores are usually nonemissive or poorly emissive at the lower concentration but are brightly emissive at the higher concentration or in the aggregated state [36,37]. Moreover, AIEE-active chemosensors offer the advantages of low background noise and a large Stokes shift [35,38]. Thus, the development of rapid, low-cost, and AIEE-active chemosensors that can selectively and sensitively detect Al^3+^ and Ga^3+^ ions as well as Asp is of utmost importance [39,40,41].

In this work, we present two isomeric quinoline- and anthracene-based Schiff bases **L1** and **L2** as the ‘turn-on’ fluorescent chemosensors. **L1** displays the AIEE properties in a MeOH-H_2_O system while also acting as a sensitive, selective, and fluorescent chemosensor for the detection of Al^3+^ and Ga^3+^ ions and Asp. In contrast, **L2**, a positional isomer of **L1**, was exclusively selective for the Ga^3+^ ion and can be used as a discriminative tool for the detection of Ga^3+^ ion even in the presence of Al^3+^ ion. The sensing abilities of **L1** were further utilized to fabricate low-cost detection methods for both Al^3+^ and Ga^3+^ ions and Asp. It is important to note that very few reports are available in the literature where an AIEE active chemosensor can detect Al^3+^ and Ga^3+^ ions and Asp [31].

## 2. Materials and Methods

### 2.1. Synthesis and Characterization 

#### 2.1.1. 2-(Anthracen-9-ylmethoxy)-3-methoxybenzaldehyde (**L’**)

*Ortho*-Vanillin (500 mg, 3.28 mmol) was taken in a round-bottomed flask (RBF) and dissolved in 5 mL of *N*,*N*-dimethyl formamide (DMF). To this solution, CsCO_3_ (1.07 g, 3.28 mmol) was added, and the mixture was stirred at room temperature for 30 minutes. Subsequently, 9-(chloromethyl)anthracene (745 mg, 3.38 mmol) was added and the reaction was stirred at room temperature for 12 h. Ice-cold water was added to this mixture, which resulted in an instantaneous thick off-white precipitate. This precipitate was filtered, thoroughly washed with water, and then dried under a vacuum. Yield: 1030 mg (92%). FTIR spectrum (Zn-Se ATR, selected peaks): 2855 (aldehyde C-H str.), 1688 (ν_C=O_) cm^−1^. ^1^H NMR spectrum (400 MHz, CDCl_3_): δ 9.99 (s, 1H), 8.52 (s, 1H), 8.42 (d, J = 8.8 Hz, 1H), 8.03 (d, J = 8.4 Hz, 1H), 7.51 (dt, J = 14.6, 6.8 Hz, 1H), 7.34 (d, J = 7.7 Hz, 1H), 7.25 (s, 1H), 7.15 (t, J = 7.9 Hz, 1H), 6.24 (s, 1H), 4.10 (s, 1H). ^13^C NMR spectrum (101 MHz, CDCl_3_): δ 190.22, 153.37, 151.53, 131.45, 131.27, 130.50, 129.34, 129.19, 127.08, 126.72, 125.11, 124.37, 123.87, 119.22, 117.98, 67.98, 56.29. ESI^+^ mass spectrum (CH_3_OH): calcd. 365.11 for C_23_H_18_O_3_ + Na^+^ (**L’** + Na^+^); obsd. 365.11 for **L’** + Na^+^.

#### 2.1.2. (*E*)-1-(2-(Anthracen-9-ylmethoxy)-3-methoxyphenyl)-*N*-(quinolin-3-yl)methanimine (**L1**)

**L’** (150 mg, 0.44 mmol) was dissolved in 25 ml of EtOH, and to this solution, 3-aminoquioline (63.4 mg, 0.44 mmol) was added and the mixture was refluxed at 80 °C for 30 min. After that, 400 μL of acetic acid was added and the mixture was further refluxed for 8 h. The reaction mixture was allowed to cool, which resulted in a precipitate that was filtered, was washed with EtOH, and was dried in a vacuum. Yield: 170 mg (83%). FTIR spectrum (Zn-Se ATR, selected peaks): 3047–3014 (aromatic C-H str.), 1624 (ν_C=N_) cm^−1^. ^1^H NMR spectrum (400 MHz, CDCl_3_): 8.51 (s, 1H), 8.42 (s, 1H), 8.38 (s, 2H), 8.36 (s, 1H), 8.07 (s, 1H), 7.92 (s, 2H), 7.65 (td, J = 6.7, 3.3 Hz, 1H), 7.62–7.56 (m, 2H), 7.53 (ddd, J = 8.0, 6.7, 1.1 Hz, 1H), 7.43–7.35 (m, 2H), 7.33–7.27 (m, 2H), 7.20–7.12 (m, 2H), 6.96 (d, J = 2.3 Hz, 1H), 6.22 (s, 2H), 4.15 (s, 3H). ^13^C NMR spectrum (101 MHz, CDCl_3_): δ 158.98, 153.24, 149.19, 147.87, 146.52, 144.77, 131.36, 131.26, 130.52, 129.18, 129.11, 129.09, 128.42, 128.29, 127.85, 127.42, 126.71, 126.65, 125.03, 124.60, 123.81, 122.50, 118.92, 115.39, 67.41, 56.27. ESI^+^ mass spectrum (CH_3_OH): calcd. 469.18 for C_32_H_24_N_2_O_2_ + H^+^ (**L1** + H^+^); obsd. 469.19 for **L1** + H^+^.

#### 2.1.3. (*E*)-1-(2-(Anthracen-9-ylmethoxy)-3-methoxyphenyl)-*N*-(quinolin-5-yl)methanimine (**L2**)

Chemosensor **L2** was synthesized using a procedure similar to that used for **L1** but with the following reagents: **L’**, (150 mg, 0.44 mmol) and 5-aminoquioline (63.4 mg, 0.44 mmol). Yield: 160 mg (78%). FTIR spectrum (Zn−Se ATR, selected peaks): 1617 (ν_C=N_) cm^−1^. ^1^H NMR spectrum (400 MHz, CDCl_3_): δ 8.91 (s, 1H), 8.41 (d, *J* = 8.4 Hz, 1H), 8.35 (dd, *J* = 6.5, 3.5 Hz, 2H), 8.32 (s, 1H), 8.31 (s, 1H), 7.85 (dd, *J* = 6.3, 3.2 Hz, 3H), 7.67 (dd, *J* = 6.5, 2.9 Hz, 1H), 7.39–7.32 (m, 6H), 7.20–7.14 (m, 2H), 6.22 (s, 2H), 6.03 (dd, *J* = 7.3, 0.8 Hz, 1H), 4.16 (s, 3H). ^13^C NMR spectrum (101 MHz, CDCl_3_): δ 157.63, 153.31, 150.48, 148.96, 148.74, 148.31, 132.72, 131.34, 131.29, 130.88, 129.39, 129.12, 129.00, 127.34, 126.58, 124.97, 124.50, 124.41, 123.85, 120.42, 119.00, 115.12, 112.85, 67.24, 56.29. ESI^+^ mass spectrum (CH_3_OH): calcd. 469.18 for C_32_H_24_N_2_O_2_ + H^+^ (**L2** + H^+^); obsd. 469.19 for **L2** + H^+^.

### 2.2. General Information and Methods

Reagents of analytical grade were procured from Sigma-Aldrich, Alfa-Aesar, and Spectrochem and were used without further purification. The solvents were purified using the standard literature methods [42]. HPLC-grade solvents were used for the UV–Visible and fluorescence spectral studies. A stock solution of **L1** and **L2** (1 mM) was prepared in Tetrahydrofuran (THF). All stock solutions of metal salts of NaCl, KCl, BeCl_2_, MgCl_2_, Al(NO_3_)_3_, GaCl_3_, Pb(NO_3_)_2_, CrCl_3_, MnCl_2_, FeSO_4_, FeCl_3_, LiCl, CoCl_2_, NiCl_2_, CuSO_4_, ZnCl_2_, AgNO_3_, Pd(CH_3_COO)_2_, Cd(NO_3_)_2_, In(OTf)_3_, and HgCl_2_ (2.5 mM) were prepared in EtOH. All stock solutions of amino acids of Glutamic acid (Glu), Proline (Pro), Cysteine (Cys), Isoleucine (Ile), Tyrosine (Tyr), Arginine (Arg), Glutamine (Gln), Lysine (Lys), Threonine (Thr), Aspartic acid (Asp), Alanine (Ala), Asparagine (Asn), Glycine (Gly), Histidine (His), Leucine (Leu), Methionine (Met), Phenylalanine (Phe), Serine (Ser), Tryptophan (Trp), and Valine (Val) were prepared in H_2_O. All UV-Vis and fluorescence spectral experiments were performed with a 1.0 cm path length cuvette at 25 °C in EtOH. 

### 2.3. Physical Measurements

FTIR spectra (Zn-Se ATR) were recorded using a PerkinElmer, USA Spectrum-Two spectrometer. NMR spectra were obtained from a JEOL, Japan 400 MHz spectrometer. High-resolution mass spectra were obtained with an Agilent G6530AA, USA (Q-TOF LC-HRMS) mass spectrometer. Fluorescence spectral studies were performed with a Cary Eclipse fluorescence spectrometer or Edinburgh Instrument, UK FLS 900 luminescence spectrometer. Time-resolved fluorescence spectra were recorded using a picosecond Fluorimeter from Horiba Jobin Yvon (FluoroHub), USA. DLS measurements were recorded on a Malvern zetasizer ZS90 instrument, UK.

### 2.4. Crystallography

X-ray diffraction intensity data of **L1** (CCDC No. 2232788) were collected at 298 K on a Bruker SMART APEX-II CCD diffractometer equipped with graphite-monochromatic Mo Kα radiation (λ = 0.71073 Å) [43]. The structure was solved using direct methods using the SIR97 program [44] and was refined on *F^2^* using all data by full-matrix least squares procedures with SHELXL 2018/3 [45]. The hydrogen atoms were placed at the calculated positions and were included in the last cycles of the refinement. All the calculations were conducted using the WinGX (ver. 2018.3) software package [46]. The crystallographic data collection and structure solution parameters are summarized in Table S1 in the Supplementary Materials.

### 2.5. DFT Studies

The geometrical optimization of chemosensors **L1** and **L2** and their complexes L1–Al^3+^, **L1**–Ga^3+^, and **L2**–Ga^3+^ was achieved under C1 symmetry by employing the density functional theory (DFT) method [47]. Becke’s three parameter exchange correlation functional by Lee, Yang, and Parr (B3LYP) was utilized as a hybrid functional for the calculations using Gaussian09W suits [48]. Two different basis sets were used for the geometrical optimization: viz., LANL2DZ for the Al^3+^ and Ga^3+^ ions and 6-311 + G (d, p) for the rest of the atoms [49,50]. The solvent effect was taken into account with a conductor-like polarizable continuum model (CPCM) using ethanol as a solvent [51]. The optimized geometries were subjected to frequency calculations to confirm if the global minima was achieved. The frontier molecular orbitals (FMOs) and molecular electrostatic potential surfaces were visualized using the optimized structures. All geometrical modifications were made using GaussView 5.0 package [52]. The FMOs were plotted using the Avogadro software [53].

### 2.6. Determination of Binding Constant (K_b_)

The binding constant (*K*_b_) was computed using the Benesi–Hildebrand Equation (1) where I, I_0_, and I_max_ are the emission intensities of the chemosensors (**L1** or **L2**) in the presence of analytes (Al^3+^, Ga^3+^, or Asp), in the absence of analytes, and the maximum fluorescence intensity in the presence of the analytes, respectively [54]. *K*_b_ was obtained by using the ratio of the intercept and slope from 1/(I − I_0_) vs. 1/[A] plot where [A] refers to the concentration of analytes, i.e., Al^3+^, Ga^3+^, or Asp.

1/(I − I_0_) = 1/{*K*_b_(I_0_ − I_max_)[A]} + 1/(I_0_ − I_max_) (1)

### 2.7. Determination of Detection Limit

The detection limit was calculated from the fluorescence spectral titration according to Equation (2), where k is the slope of a plot of the emission intensity of the chemosensors (**L1** or **L2**) versus the concentration of analytes (Al^3+^, Ga^3+^ or Asp) while σ is the standard deviation of ten blank replicate fluorescence measurements of chemosensors (**L1** or **L2**) [55,56].

Detection limit: 3σ/k(2)

### 2.8. Determination of Quantum Yield

The relative fluorescence quantum yields (Φ_F_) of **L1**, **L1** at f_w_ = 70% **L1**–Al, **L1**–Ga, **L1**–Asp, **L2**, and **L2**–Ga were calculated using Equation (3) [57,58,59]:

Φ_F_ = Φ_st_× S_u_/S_st_× A_st_/A_u_× n^2^D_u_/n^2^D_st_ (3)

where Φ_F_ is the fluorescence quantum yield of the samples. Here in the equation, the subscripts u and st refer to the unknown and standard samples, respectively. Φ_st_ refers to the fluorescence quantum yield of the Quinine sulfate (Φ_st_ = 0.54) used as the standard sample here. A_u_ and A_st_ are the absorbance of the unknown and standard samples, respectively. S_u_ and S_st_ refer to the integrated emission band areas of the unknown and standard samples, respectively. nD_st_ and nD_u_ represent the solvent refractive index of the standard and the unknown sample, respectively.

### 2.9. Colorimetric Analysis

For colorimetric analysis, standard solutions of assorted metal ions and amino acids (2.5 equiv.) were added to a solution of chemosensor **L1** in EtOH. Their photographs were taken under a UV lamp (λ_ex_ = 365 nm) [4,56].

### 2.10. Fabrication of Filter Paper Strips

Strips of Whatman filter paper were dipped in an ethanolic solution of chemosensor **L1** followed by drying in the open air to prepare the test strips. Such **L1** coated test strips were dipped into an aqueous solution of Al^3+^ and Ga^3+^ ions as well as Asp (2.5 equiv.). These paper test strips were then investigated under the UV lamp (λ_ex_ = 365 nm) [58,59]. 

### 2.11. Fabrication of Polystyrene Films

The chemosensor **L1** was mixed with styrene (0.5 mL) and α,α’-azoisobutyronitrile (AIBN; 1 mg) in EtOH (0.5 mL). The resultant mixture was heated in a water bath at 80 °C for 30 min. Subsequently, a few drops of the hot clear solution were poured over a glass slide and the resultant glass slide was dried in air to produce the thin films of chemosensor **L1**-loaded polystyrene. Such polystyrene films were peeled off from the glass slide and were used to detect Al^3+^ and Ga^3+^ ions as well as Asp by dipping them in their respective aqueous solutions (2.5 equiv.). These polystyrene films were then photographed under the UV lamp (λ_ex_ = 365 nm) [4,57].

## 3. Results and Discussion 

### 3.1. Synthesis and Characterization of Chemosensors ***L1*** and ***L2***

**Scheme 1:**
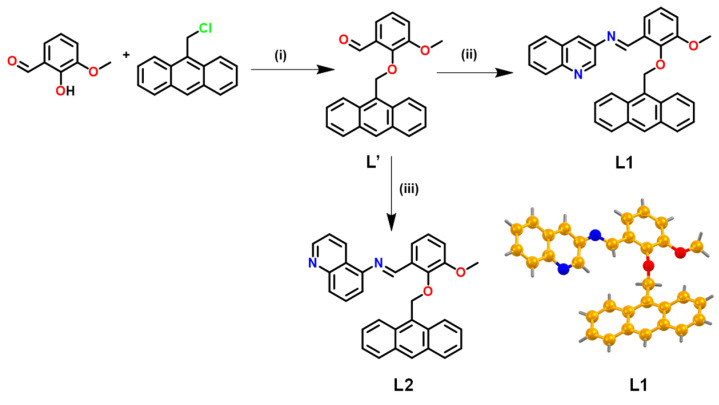
Preparative routes for the synthesis of chemosensors **L1** and **L2**. Conditions: (i) CsCO_3_, DMF, RT, 12 h; (ii) 3-aminoquinoline, EtOH, CH_3_COOH, reflux, 12 h; (iii) 5-aminoquinoline, EtOH, CH_3_COOH, reflux, 12 h. Crystal structure of **L1** in ball and stick model where carbon atoms are shown in golden color while N and O atoms are represented in blue and red colors, respectively.

An anthracene-based aldehyde, **L’**, was synthesized by reacting o-vanillin with 9-(chloromethyl)anthracene (Scheme 1). **L’** was characterized by various spectroscopic studies (Figures S1–S4). The chemosensors **L1** and **L2** were synthesized by the condensation of **L’** with 3-aminoquinoline and 5-aminoquinoline, respectively (Scheme 1). The positional isomers **L1** and **L2** were characterized by various spectroscopic techniques. Both **L1** and **L2** displayed the characteristic ν_C = N_ stretches at 1626 and 1623 cm^−1^, respectively (Figures S5 and S9). ^1^H NMR spectra of **L1** and **L2** exhibited the imine-H signal at 8.52 and 8.91 ppm, respectively (Figures S6 and S10). The ^13^C resonances for the imine-C were noted at approx. 159 and 158 ppm for **L1** and **L2**, respectively (Figures S7 and S11). The ESI^+^ mass spectra of both chemosensors **L1** and **L2** displayed a peak at m/z 469.19 corresponding to [**L1/L2** + H]^+^ (Figures S8 and S12). To further authenticate the molecular structure, **L1** was characterized by the single crystal X-ray diffraction technique (Scheme 1). **L1** crystallized in a centrosymmetric space group C2/c. Notably, arene, quinoline, and anthracene rings were making different angles with each other due to the electron cloud repulsion and thus created a cavity to potentially accommodate a suitable analyte.

### 3.2. Photophysical Properties of ***L1*** and ***L2***

The absorption and emission spectra of both **L1** and **L2** (c, 20 μM) were recorded in EtOH. The absorption spectrum of **L1** exhibited two intense high-energy bands at 288 nm (ε = 45,765 M^−1^ cm^−1^) and 333 nm (ε = 47,500 M^−1^ cm^−1^) corresponding to π→π* and n→π* transitions, respectively, with shoulders at 365 nm (ε = 29,270 M^−1^ cm^−1^) and 386 nm (ε = 16,645 M^−1^ cm^−1^ (Figure S13) [56,60,61]. The absorption spectrum of **L2** displayed a high energy band at 348 nm (ε = 42,500 M^−1^ cm^−1^) along with various shoulders at 335 (ε = 41,000 M^−1^ cm^−1^), 367 (ε = 32,500 M^−1^ cm^−1^), and 387 nm (ε = 21,000 M^−1^ cm^−1^) (Figure S13). The emission behavior of **L1** and **L2** was examined at different excitation wavelengths (320–370 nm); however, the maximum emission intensity for both **L1** (at 410 nm) and **L2** (at 430 nm with shoulders at 408 and 460 nm) was observed when excited at λ_ex_ = 350 nm (Figure S14). Thus, all subsequent studies were conducted at an excitation wavelength of 350 nm for both chemosensors.

### 3.3. AIEE Studies

**Figure 1:**
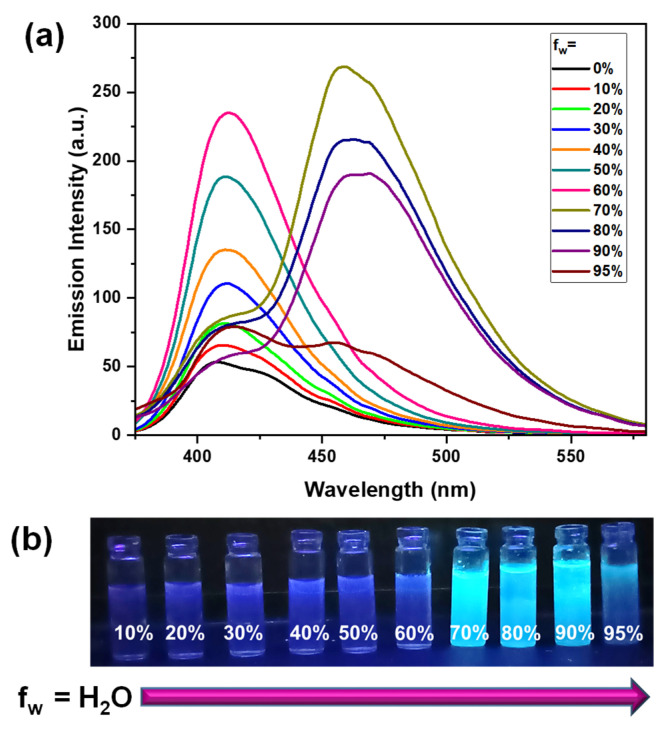
(**a**) Emission spectra of **L1** (c, 50 µM, λ_ex_ = 350 nm) in a MeOH-H_2_O mixture with water fraction increasing from 0–95%; (**b**) optical images of vials containing **L1** in MeOH with water fraction increasing from 10–95% under the UV illumination.

**Figure 2:**
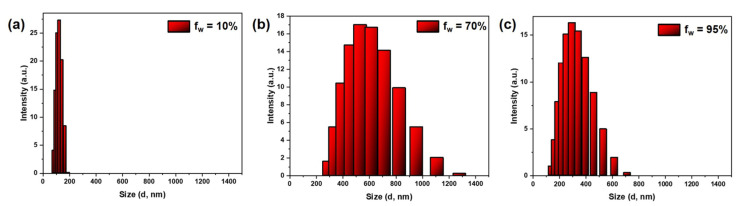
DLS measurements of **L1**-aggregates formed in (**a**) 10, (**b**) 70, and (**c**) 95% MeOH–water solvent system.

Both **L1** and **L2** were soluble in various polar and nonpolar solvents such as MeOH, EtOH, THF, CHCl_3_, DCM, DMF, and DMSO but were insoluble in water. Therefore, in order to induce AIEE, the optical properties of **L1** and **L2** were examined in MeOH (c, 50 μM) after adding different percentages of water (f_w_ = 0 to 95%). In fluorescence spectra, as the percentage of water (f_w_, %) increased from 0 to 60 in a MeOH solution of **L1**, the emission intensity increased constantly at 411 nm and attained a maxima at this wavelength (Figure 1a). This enhancement is attributed to the aggregation of **L1** [32,38]. Notably, as the water percentage was further increased to 70%, a significant bathochromic shift to 460 nm (∆λ = 50 nm) was observed. Furthermore, when the water percentage was further increased, the emission intensity decreased but without any shift in wavelength. At f_w_ = 95%, the emission bands were distributed equally at both wavelengths. We believe as the hydrophobic anthracene rings repel water, they minimize their contact with water and thus rupture the aggregates beyond a water percentage of 70% [62,63]. Figure 1b displays changes at various percentages of water varying from 10% to 95% in a MeOH-H_2_O system under UV light. Notably, the emission spectrum of **L2** did not display any change with different percentages of water and hence **L2** was AIEE inactive (Figure S15) [32]. In the case of **L1**, aggregate formation was confirmed by the dynamic light scattering (DLS) studies (Figure 2). DLS measurements revealed that the average diameter of the aggregates increased from 118 nm at f_w_ = 10% to 610 nm at f_w_ = 70%. However, at f_w_ = 95%, the size of aggregates decreased to 293 nm. These results thus confirm that not only does **L1** form aggregates in the presence of water, but maximum aggregation was possible at f_w_ = 70%.

Time-resolved fluorescence spectroscopy was employed to further confirm AIEE and aggregate formation in **L1**. Since lifetime measurements are independent of the excitation wavelength and concentration, such studies are more conclusive [64]. The fluorescence lifetime (τ_av_) increased from 3.38 ns at f_w_ = 0 % to 5.73 ns at f_w_ = 70%, implying the aggregate formation (Figure S16 and Table S2). However, τ_av_ decreased to 4.47 ns at f_w_ = 95%, further confirming that the maximum aggregation was possible at f_w_ = 70%. To obtain further support, the fluorescence quantum yield (Φ_F_) was calculated for **L1**. The quantum yield was very low (Φ_F_ = 0.008) at f_w_ = 0%; however, it increased nearly four-fold at f_w_ = 70% (Φ_F_ = 0.031). As expected, Φ_F_ again decreased to 0.023 at f_w_ = 95%, confirming the formation of maximum aggregates only at f_w_ = 70%.

**Scheme 2:**
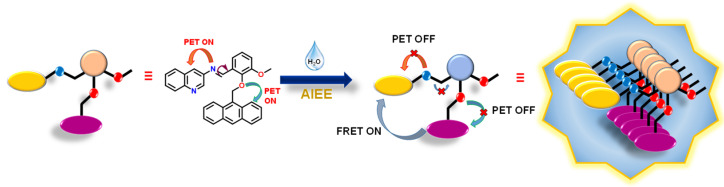
Proposed AIEE mechanism exhibited by **L1**.

For **L1**, both the low quantum yield and lifetime at f_w_ = 0% suggested the operation of Photoinduced Electron Transfer (PET) from imine-N and phenolic-OR (R: -CH_2_-) to the quinoline and anthracene moieties, respectively (Scheme 2). In addition, the intramolecular rotation of the imine-C bond further quenched the emission intensity via nonradiative decay at f_w_ = 0%. However, as water was added, both imine-N and phenolic-OR groups started forming H-bonds with the water molecule(s), which in turn subdued PET and restricted the intramolecular bond rotation [32,65,66]. Moreover, fluorescence resonance energy transfer (FRET) also occurred from the anthracene ring to the quinoline ring [67]. As a result, aggregation occurred, and the emission intensity increased.

### 3.4. Detection of Al^3+^ and Ga^3+^ Ions by UV-Vis Spectral Studies

**Figure 3:**
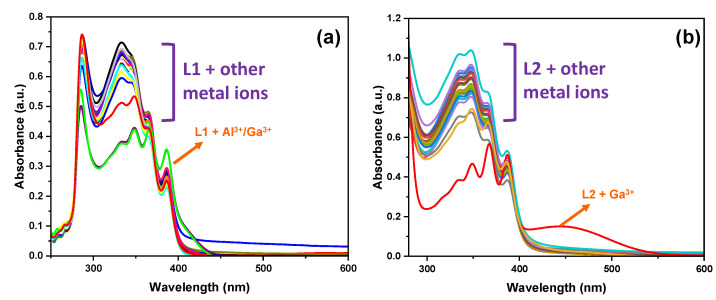
Absorption spectra of chemosensors (**a**) **L1** (c, 20 μM) and (**b**) **L2** (c, 20 μM) in EtOH in the presence of assorted metal ions (c, 50 μM).

To understand the detection ability of **L1** and **L2**, absorption spectra of **L1** (c, 20 μM) and **L2** (c, 20 μM) were examined in the presence of 2.5 equiv. of assorted metal ions (Ag^+^, Cd^2+^, Mn^2+^, Zn^2+^, Hg^2+^, K^+^, Na^+^, Co^2+^, Pd^2+^, Mg^2+^, Pb^2+^, Be^2+^, Ni^2+^, Sn^2+^, Li^+^, Cr^3+^, Fe^2+^, Fe^3+^, Cu^2+^, Al^3+^, Ga^3+^, In^3+^) (Figure 3). The absorption spectra of **L1** did not show prominent changes with any of the metal ions except the Al^3+^ and Ga^3+^ ions. In contrast, the absorption spectra of **L2** displayed a significant change only in the presence of Ga^3+^ ions. During the absorption spectral titration of **L1**, the spectral bands at 333, 365, and 385 nm were altered with an isosbestic point at 360 nm, suggesting the formation of a new species (Figure S17). In the case of **L2**, the band at 348 nm decreased while two new bands appeared at 270 and 465 nm with an isosbestic point at 283 nm [56]. These spectral titrations supported the formation of a complex between the chemosensors and metal ions.

### 3.5. Detection of Al^3+^ and Ga^3+^ Ions by Fluorescence Spectral Studies

**Figure 4:**
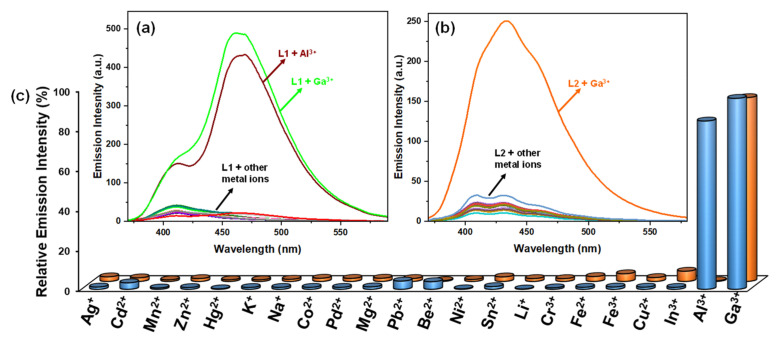
Emission spectra of chemosensors (**a**) **L1** (c, 20 μM) and (**b**) **L2** (c, 20 μM) in EtOH in the presence of assorted metal ions (c, 50 μM). (**c**) Bar diagram showing the relative change in the emission intensity of **L1** (blue bars) and **L2** (orange bars) in the presence of assorted metal ions (c, 50 μM).

The emission spectra of both chemosensors **L1** (c, 20 μM) and **L2** (c, 20 μM) were measured in the presence of 2.5 equiv. of different metal ions: Ag^+^, Cd^2+^, Mn^2+^, Zn^2+^, Hg^2+^, K^+^, Na^+^, Co^2+^, Pd^2+^, Mg^2+^, Pb^2+^, Be^2+^, Ni^2+^, Sn^2+^, Li^+^, Cr^3+^, Fe^2+^, Fe^3+^, Cu^2+^, Al^3+^, Ga^3+^, and In^3+^ (Figure 4). In the case of **L1**, the emission spectral profile remained unchanged in the presence of nearly all the metal ions except for the Al^3+^ and Ga^3+^ ions. It is significant to note that **L1** showed a ‘turn-on’ response in the presence of both Al^3+^ and Ga^3+^ ions with the observation of a new emission band at 465 nm. In contrast, **L2** displayed an enhanced emission at 430 nm only in the presence of Ga^3+^ ions while no change was noted with the Al^3+^ ion as well as other metal ions [6]. Therefore, the chemosensor **L2** was able to discriminate between the Al^3+^ and Ga^3+^ ions. The bar diagram nicely illustrates the change in the emission intensity of **L1** and **L2** after the addition of different metal ions (Figure 4c). It is important to mention that both chemosensors did not exhibit any spectral change with the In^3+^ ion.

**Figure 5:**
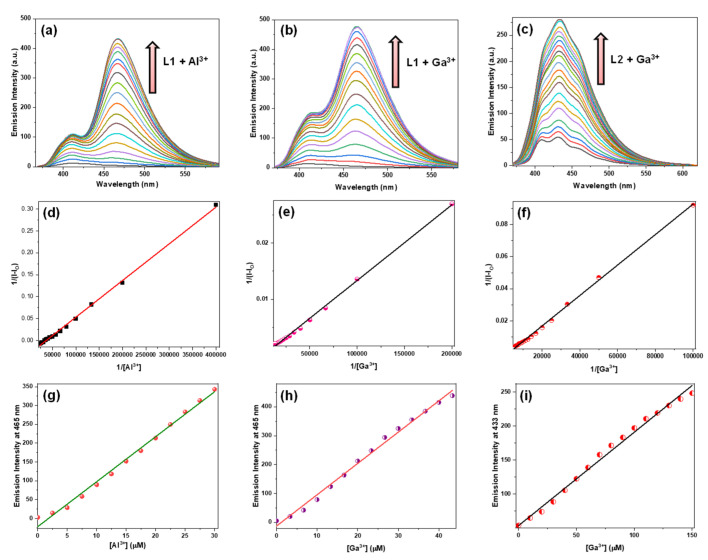
Change in the emission spectra of **L1** (c, 20 μM) on successive addition of (**a**) Al^3+^ ion (0–75 μM) and (**b**) Ga^3+^ ion (0–75 μM); (**c**) **L2** (c, 20 μM) on successive addition of Ga^3+^ ion (0–200 μM). Determination of binding constants by Benesi–Hildebrand plots for the detection of (**d**) Al^3+^ ion, (**e**) Ga^3+^ ion by **L1**, and (**f**) Ga^3+^ ion by **L2** from the emission spectral profiles. Determination of detection limit of **L1** towards the (**g**) Al^3+^ ion, (**h**) Ga^3+^ ion, and (**i**) **L2** towards the Ga^3+^ ion. All studies were carried out in EtOH.

To determine the extent of binding, emission spectral titrations were performed (Figure 5a–c). From the concentration variation plots, binding constants (Figure 5d–f) and detection limits (Figure 5g–i) were calculated by using the linear fitting methods. **L1** displayed high binding constants of 3.58 × 10^4^ and 2.80 × 10^4^ M^−1^ for the Al^3+^ and Ga^3+^ ions, respectively [54,68]. Further, **L1** exhibited remarkable detection limits of 0.19 and 0.22 μM for the Al^3+^ and Ga^3+^ ions, respectively [69]. On the other hand, **L2** displayed a detection limit of 1.31 μM and a binding constant of 2.24 × 10^3^ M^−1^ towards the Ga^3+^ ion, which was impressive but a little inferior to that of **L1**. Importantly, both the detection limit and linear range of the metal ions exhibited by the present chemosensor were better than that of other chemosensors reported in the literature (Table S3).

### 3.6. Selectivity, Lifetime, and Quantum Yield Measurements

**Figure 6:**
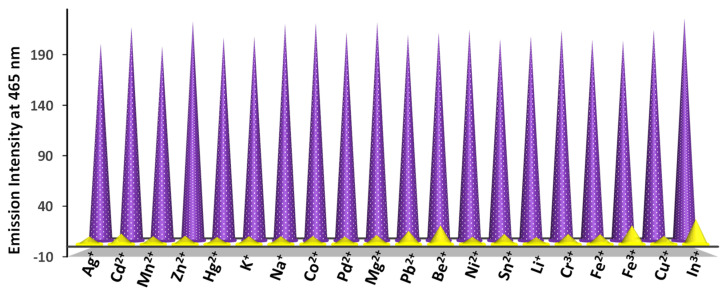
Selectivity of chemosensor **L1** towards the Al^3+^ ion in the presence of other metal ions: **L1** + metal ions (yellow cones) and **L1** + metal ions + Al^3+^ ion (purple cones). All studies were conducted in EtOH.

For a chemosensor, selectivity towards a particular analyte is crucial as a chemosensor may have to identify a specific analyte in a competing environment [70,71]. Thus, the selectivity of **L1** and **L2** towards Al^3+^/Ga^3+^ ions in the presence of other competing metal ions was attempted by studying the competitive binding studies (Figure 6 and Figure S18). The emission spectra of **L1** and **L2** were recorded with Al^3+^/Ga^3+^ ions in the presence of the equimolar concentration of other metal ions. Both for **L1** and **L2**, no measurable change in the emission intensity was observed towards the detection of Al^3+^/Ga^3+^ ions in the presence of different metal ions including In^3+^ ions. Notably, for **L2**, the emission intensity was unaffected even in the presence of Al^3+^ ions. It is, therefore, evident that both **L1** and **L2** were highly selective for Al^3+^/Ga^3+^ ions even in the presence of assorted metal ions.

**Figure 7:**
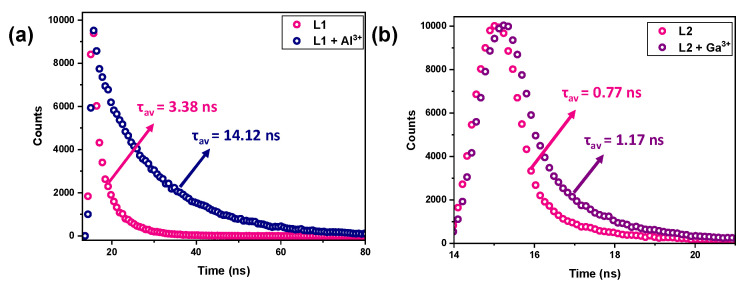
Lifetime profiles of chemosensors (**a**) **L1** and (**b**) **L2** in the absence and presence of Al^3+^ and Ga^3+^ ions, respectively, in EtOH.

The excitation energy of a chemosensor may change after its interaction with an analyte. Hence, the excited state behavior of **L1** and **L2** in the absence and presence of Al^3+^/Ga^3+^ ions was evaluated through the lifetime measurements [70]. **L1** exhibited a biexponential decay with an average lifetime (τ_av_) of 3.38 ns. In the presence of Al^3+^ and Ga^3+^ ions, τ_av_ increased to 14.12 and 13.62 ns, respectively (Figure 7a, Figure S19, and Table S2). A similar four-fold enhancement in τ_av_ of **L1** in the presence of Al^3+^ and Ga^3+^ ions suggested that both these ions offered a similar mode of interaction with **L1**. However, **L2** displayed a triexponential decay with τ_av_ of 0.77 ns, which increased 1.5-fold times to 1.17 ns in the presence of Ga^3+^ ions (Figure 7b). The quantum yields of **L1** and **L2** were also measured in the absence and presence of Al^3+^/ Ga^3+^ ions by taking Quinine sulfate (Φ_F_ = 0.54) as a reference [55]. The resultant Φ_F_ for **L1**, **L1**−Al^3+^, and **L1**−Ga^3+^ were found to be 0.008, 0.065, and 0.062, respectively. Thus, enhancement in the quantum yield was approximately eight-fold both for the Al^3+^ and Ga^3+^ ions. The calculated Φ_F_ for **L2**−Ga^3+^ (0.010) displayed a 2.5-fold enhancement when compared to **L2** (0.003). These results further support the fluorescence lifetime studies. Collectively, all studies suggest that the binding mechanisms of **L1** and **L2** towards the Al^3+^/Ga^3+^ ions were different.

### 3.7. Reversibility Studies

**Figure 8:**
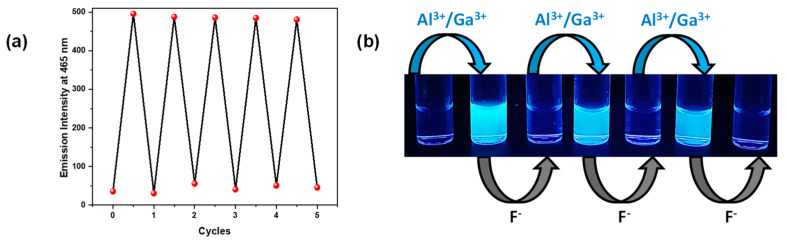
(**a**) Reversibility cycles for **L1** (c, 20 μM) upon subsequent addition of Al^3+^ ion (as Al(NO_3_)_3_, 2.5 equiv.) and F^−^ ion (as NaF, 2.5 equiv.) in EtOH. (**b**) Reversibility cycles for **L1** using Al^3+^ ion (as Al(NO_3_)_3_)/Ga^3+^ ion (as GaCl_3_) and F^−^ ion (as NaF) under the UV light.

The reversibility of a chemosensor is an essential requirement that endows it for practical applications. The reversibility of **L1**–Al^3+^ and **L1**–Ga^3+^ complexes was checked with various anions; however, the reversibility was only achieved by the addition of fluoride ions (as NaF) (Figure 8) [6]. In fact, the F^−^ ion was able to show multiples cycles of reversibility without any potential loss in the chemical and emission behavior of **L1**. Such a fact suggests that the F^−^ ion removed the Al^3+^ and Ga^3+^ ions from their metal complexes while generating free **L1** and were ready for the next round of detection of Al^3+^ and Ga^3+^ ions.

### 3.8. Mode of Binding 

**Scheme 3:**
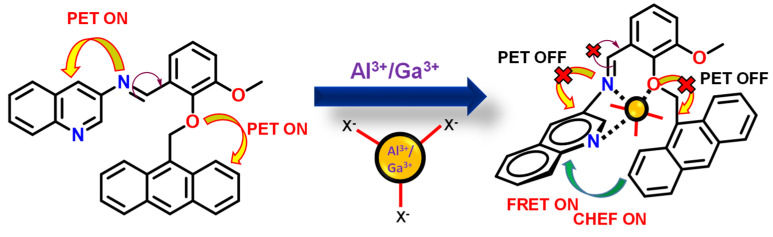
Sensing mechanism of **L1** towards Al^3+^/Ga^3+^ ions via fluorescence ‘turn-on’ strategy.

The absorption and emission spectral studies confirmed the interaction and/or binding of Al^3+^/Ga^3+^ ions to **L1** and **L2**. Both chemosensors **L1** and **L2** offer three types of functional groups: phenolic-OR (R = -CH_2_- or -CH_3_), imine-N, and quinoline-N. Both Al^3+^ and Ga^3+^ ions are hard acids and are thus likely to interact and/or bind preferentially with the phenolic-OR followed by imine-N [4,14]. For positional isomers **L1** and **L2**, the position of the quinoline-N is likely to play a crucial role. In the case of **L1**, the binding of a metal ion through phenolic-OR (R = -CH_2_-), imine-N, and quinoline-N results in the formation of both six- and five-membered chelate rings and supports chelation-enhanced fluorescence. It is important to mention that a metal ion (Al^3+^/Ga^3+^) is situated significantly above (approx. 2.7–2.8 Å) the plane of **L1** to facilitate the simultaneous coordination of phenolic-OR, imine-N, and quinoline groups, as depicted by the DFT studies (vide infra). Due to the formation of such chelate rings, **L1** achieves structural rigidity while the intramolecular bond rotation of the imine-N group becomes restricted (Scheme 3). At the same time, excited-state electron transfer from imine-N and phenolic-OR to quinoline rings and anthracenyl moieties also gets inhibited. Collectively, these points suppress the PET [72,73]. Moreover, as PET is subdued, the fluorescence resonance energy transfer (FRET) takes place between the anthracene and quinoline rings [2]. Therefore, as a result of the turning off of the PET process and turning-on of CHEF and FRET, the emission intensity of **L1** increases in the presence of Al^3+^/Ga^3+^ ions.

**Figure 9:**
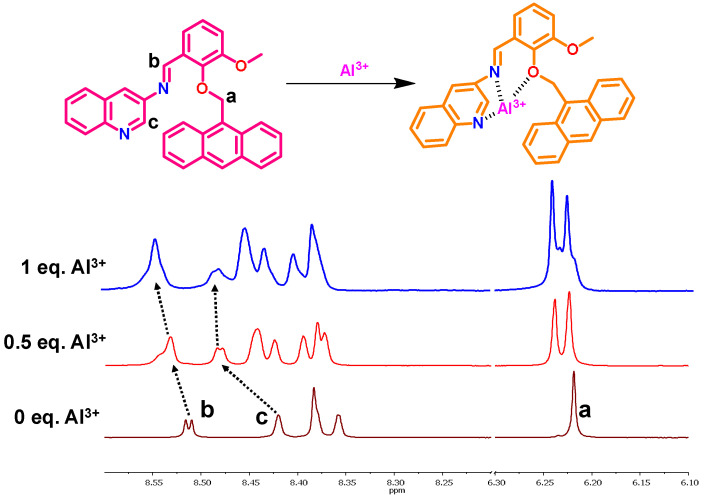
Selected part of ^1^H NMR spectra displaying the titration of **L1** with the Al^3+^ ion (0–1 equiv.) in CDCl_3_. See Figure S20 for full-range spectrum.

All studies pointed to a similar mode of binding of both Al^3+^ and Ga^3+^ ions with **L1**. To further confirm the binding mode of **L1**, an ^1^H NMR spectral titration with Al^3+^ ions was performed (Figure 9 and Figure S20). When sequentially adding one equivalent of Al^3+^ ions to a solution of **L1**, imine-N, phenolic-OR (R = -CH_2_-), and quinoline-N were shifted downfield, confirming their involvement in binding to the Al^3+^ ion. Moreover, the quinoline ring protons also shifted downfield, thus further confirming their role in binding. Importantly, both -CH_2_- and -CH_3_ protons were completely resolved, indicating a difference in their chemical environment after the binding of the Al^3+^ ion. Further, anthracene ring protons were also perturbed after the potential coordination of an Al^3+^ ion. Notably, various aromatic protons were found to split to give multiplets. We propose that imine-N, phenolic-OR, and quinoline-N form a pocket in which a metal ion nicely resides. Such a binding mode affects the chemical environment of nearby arene rings and hence multiplets were observed [4].

In **L2**, the position of quinoline-N was changed, which is now anti to the imine-N group. As a result, the formation of a common binding pocket was not possible. It is therefore proposed that a larger Ga^3+^ ion is able to interact with both phenolic-OR (R = -CH_2_-, -CH_3_) groups and imine-N. As the Al^3+^ ion is smaller in size, it could not interact with all three groups, and hence **L2** did not show its recognition. The enhanced emission was observed due to the suppression of PET and ‘turn-on’ of CHEF. As the quinoline ring was not involved in binding to a Ga^3+^ ion, FRET was not operative [74,75]. This proposition was further confirmed by the ^1^H NMR spectral studies where protons of both phenolic-OR and imine-N displayed a downfield shift whereas both quinoline and anthracene rings remained unaffected (Figure S21).

To characterize the actual species formed between **L1** and Al^3+^/Ga^3+^ ions, ESI^+^ mass spectra were recorded. The mass spectra of **L1**−Al^3+^ species displayed a peak at m/z 673.27 which corresponded to [**L1** + Al^3+^ + 2NO_3_^—^ + OCH_3_^—^ + Na^+^]^+^. On the other hand, **L1**−Ga^3+^ species showed a peak at m/z 669.30 corresponding to [**L1** + Ga^3+^ + 3OCH_3_^−^ + K^+^]^+^ (Figures S22 and S23). The sources of the NO_3_^−^ and OCH_3_^−^ ions were from an aluminum precursor, Al(NO_3_)_3_, and a CH_3_OH solvent, respectively. It is important to note that, for both species, the isotopic distribution patterns nicely matched that of the simulated patterns. The mass spectra further asserted a 1:1 metal binding stoichiometry to **L1** [58]. The said binding stoichiometry was further confirmed by Job’s plot for both Al^3+^ and Ga^3+^ ions (Figure S24) [59].

### 3.9. DFT Studies

**Figure 10:**
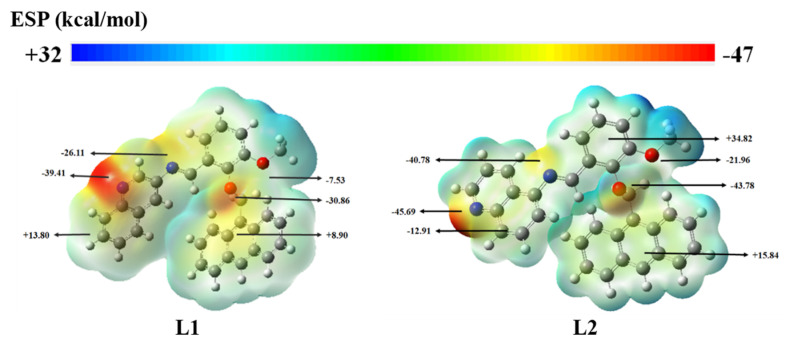
Electrostatic potential maps with the approximate potentials (in kcal/mol) across the marked regions.

All chemical structures were optimized by the DFT using the suitable basis sets and functional (see Table S4 for the Cartesian coordinates). The molecular electrostatic potential surface (MESP) maps are often employed for the qualitative investigation of the charge distribution in a system. Hence, the optimized structures of chemosensors **L1** and **L2** were used to study the electropositive and electronegative centers. As shown in Figure 10, the electrostatic potential was scaled between −47.0 and +32.0 kcal/mol for both **L1** and **L2** with their approximate values marked. Notably, both oxygen and nitrogen centers in **L1** and **L2** were relatively more electronegative as depicted from the red-colored region around them. The MESP maps therefore suggest that the metal ions can bind with these groups: quoniline-N, imine-N, and phenolic-OR (-CH_2_-).

**Figure 11:**
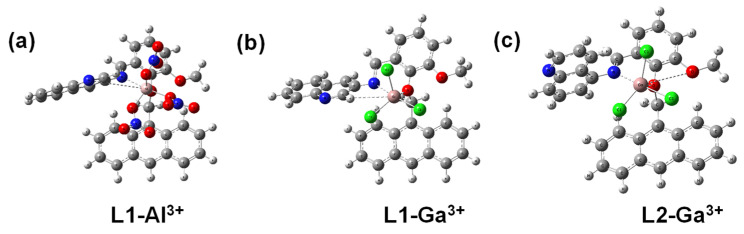
Optimized structures of (**a**) **L1**-Al^3+^, (**b**) **L1**–Ga^3+^, and (**c**) **L2**–Ga^3+^ complexes (Isovalue = 0.02). The associated anions (NO_3_^−^ and Cl^−^) are shown in their usual colors.

The experimental results show that **L1** exhibited FRET with both Al^3+^ and Ga^3+^ ions; however, FRET was not observed for **L2** with the Ga^3+^ ion. In order to obtain insights about the differences in their binding modes, the structures of chemosensor–metal complexes were optimized (Figure 11). The DFT optimized structures of **L1**–Al^3+^, **L1**–Ga^3+^, and **L2**–Ga^3+^ were in line with the experimental results. Notably, in the case of **L1**, both the Al^3+^ and Ga^3+^ ions were situated significantly above (approx. 2.7–2.8 Å) the plane of the chemosensor to facilitate the simultaneous coordination of all binding sites. The optimized structure of **L2**–Ga^3+^ confirmed the involvement of the phenolic-OCH_3_ group (ESP: −21.96 kcal/mol) in binding in addition to the phenolic-OCH_2_- group. In contrast, quinoline-N was situated farthest at 6.19 Å from the Ga^3+^ ion and thus remained uncoordinated. To better understand the electron density shifts, contour plots of the frontier molecular orbitals (FMOs) from HOMO-4 to LUMO+4 were visualized (Figure S25; see Figure S26 for a magnified view of the HOMO-1 to LUMO+1 orbitals). The electron density was found to be localized on the anthracene ring in HOMO but shifted to the quinoline ring in LUMO. In addition, the HOMO-LUMO band gap energy for **L1** (3.42 eV) and **L2** (3.64 eV) decreased to 2.42, 2.85, and 3.44 eV corresponding to **L1**–Al^3+^, **L1**–Ga^3+^, and **L2**–Ga^3+^, respectively (see Table S5 for energy values (in eV) for all FMOs). Thus, a decrease in the energy gaps upon metal complexation further supports the chemosensing behavior of these chemosensors.

### 3.10. Detection of Aspartic Acid 

**Figure 12:**
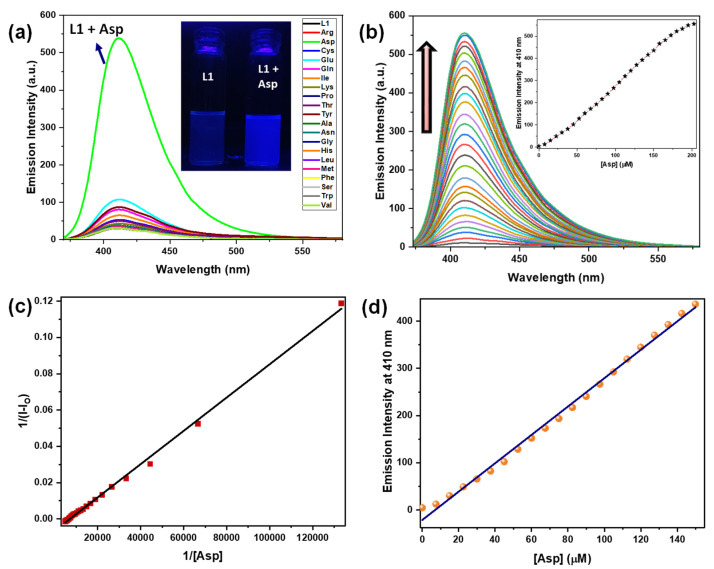
(**a**) Emission spectra of chemosensor **L1** (c, 20 μM) in the presence of assorted amino acids. Inset displays a vial containing **L1** and **L1**+Asp under the UV lamp illumination. (**b**) Change in the emission spectra of **L1** (c, 20 μM) on successive addition of Asp. (**c**) Determination of the binding constant by Benesi–Hildebrand plot for the detection of Asp by **L1** from the emission spectral titration. (**d**) Determination of detection limit of **L1** towards Asp. All studies were conducted in EtOH.

The recognition of amino acids is challenging as they contain both carboxylic acid and amino groups [24,76]. Therefore, designing a chemosensor that can selectively detect an amino acid is always difficult [77]. Chemosensors **L1** and **L2** containing electron-rich groups and various binding sites make them ideal for the detection of amino acids [27,29]. Hence, the detection ability of **L1** and **L2** was checked towards all 20 amino acids: Gly, Ala, Glu, Pro, Cys, Ile, Tyr, Arg, Gln, Lys, Thr, Asp, Asn, His, Leu, Met, Phe, Ser, Trp, and Val. Notably, for **L1**, the emission intensity increased only in the presence of Asp at 410 nm. In contrast, **L2** did not display any change in its emission spectra with any of the amino acids (Figure 12a and Figure S27). It is thus clear that chemosensor **L1** is able to selectively detect Asp. Subsequently, an emission spectral titration of **L1** was performed after the incremental addition of Asp from 0–200 μM (Figure 12b). From the concentration variation plot, the binding constant and limit of detection were found to be 2.89 × 10^3^ M^−1^ and 0.80 μM, respectively (Figure 12c,d) [78].

**Figure 13:**
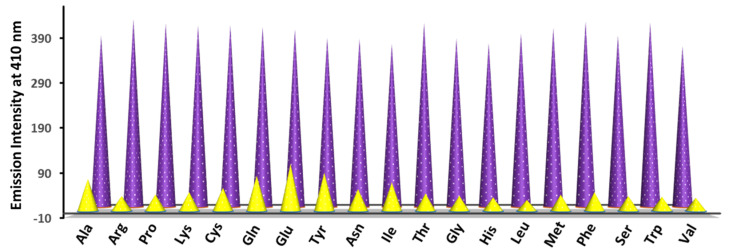
Selectivity of chemosensor **L1** towards Asp in the presence of other amino acids: **L1** + amino acids (yellow cones) and **L1** + amino acids + Asp (purple cones). All studies were conducted in EtOH.

The selectivity of **L1** towards Asp was evaluated by the competitive binding studies in the presence of an equimolar amount of all 20 amino acids [28]. The emission intensity of **L1**−Asp at 410 nm remained unaffected in the presence of other amino acids (Figure 13). The excited state behavior of **L1** was studied in the presence of Asp with the lifetime measurement. With Asp, **L1** displayed a biexponential decay with an average lifetime (τ_av_) of 5.09 ns with a nearly 1.5-fold enhancement (Figure S28 and Table S2). The quantum yield of **L1**−Asp was found to be 0.027 with an approx. three-fold enhancement. Therefore, enhancement both in the lifetime measurement and quantum yield in the presence of Asp supports the interaction of **L1** with Asp [57].

To identify the binding sites, an ^1^H NMR spectrum of **L1** was recorded in the presence of 1 eq. of Asp. The imine-N and phenolic-OR (R = -CH_2_- and -CH_3_) protons displayed a downfield shift (Figure S29). Notably, a small downfield shift in the quinoline ring protons was also noted, suggesting that the quinoline-N was interacting with Asp. Such an interaction aligns with both **L1** and Asp to maximize their interaction. However, the aromatic ring protons, i.e., anthracene and arene, remained unaffected. These results suggest that imine-N, both phenolic-OR groups, and quinoline-N are the potential interacting sites of **L1** towards a molecule of Asp [29]. We propose that due to such interactions, PET in **L1** was suppressed, resulting in an enhanced emission. Importantly, Asp was not recognized by **L2** because of the fact that the quinoline-N group was farthest located, which did not allow it to interact with Asp to achieve the correct conformation and hence its sensing was not observed.

### 3.11. Low-Cost Detection Methods 

**Figure 14:**
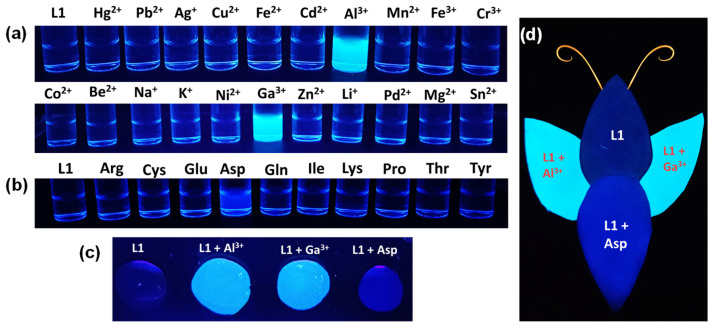
Optical images of chemosensor **L1** (**a**) with different metal ions (2.5 equiv.) and (**b**) amino acids (2.5 equiv.) in solution state; (**c**) polystyrene films loaded with **L1** in the absence and presence of Al^3+^ and Ga^3+^ ions and Asp. (**d**) Filter paper test strips of **L1** in the absence and presence of Al^3+^ and Ga^3+^ ions and Asp. All images were taken under a UV lamp (λ_ex_ = 365 nm).

The ‘turn-on’ detection of Al^3+^ and Ga^3+^ ions by both chemosensors prompted us to attempt different low-cost detection methods. As the best sensing results were observed for **L1**, low-cost detection methods were explored using the chemosensor **L1** (Figure 14). The colorimetric detection was attempted by introducing different metals (c, 2.5 mM) and amino acids (c, 2.5 mM) to an EtOH solution of **L1**. Notably, **L1** displayed detectable enhanced emission under UV light (λ_ex_ = 365 nm) with both Al^3+^ and Ga^3+^ ions and Asp when compared to other metals and amino acids [79]. Next, polystyrene films were prepared containing the chemosensor **L1 [4]**. Such peelable polystyrene films displayed enhanced emission under UV light for the detection of both Al^3+^ and Ga^3+^ ions as well as Asp from their aqueous solutions. Similarly, filter paper test strips containing **L1** were able to detect both Al^3+^ and Ga^3+^ ions (c, 2.5 mM) and Asp (c, 2.5 mM) from the aqueous solutions of different metal ions and amino acids [56]. Importantly, such filter paper test strips, tested with different concentrations of Al^3+^ ions (0, 5, 20, and 50 μM), were able to detect concentrations as low as 5 μM by showing color change from blue to cyan under the UV lamp (Figure S30). Hence, low-cost detection methods can be utilized to detect both Al^3+^ and Ga^3+^ ions as well as Asp for practical on-site detections.

## 4. Conclusions

This work illustrates two Schiff-base-based chemosensors, **L1** and **L2**, decorated with electron-rich quinoline and anthracene rings. Chemosensor **L1** displayed AIEE in a MeOH-H_2_O solvent system due to the suppression of PET. DLS measurements and fluorescence lifetime studies confirmed the aggregation of **L1** in the presence of water. Chemosensor **L1** was utilized for the sensitive, selective, and reversible detection of Al^3+^ and Ga^3+^ ions in the presence of other metal ions. **L1** displayed ‘turn-on’ emission in the presence of both Al^3+^ and Ga^3+^ ions with high binding constants and low detection limits. Chemosensor **L2**, a structural isomer of **L1**, was able to discriminate between the Al^3+^ and Ga^3+^ ions and was exclusively selective for the Ga^3+^ ion with an impressive detection limit and a high binding constant. The discriminative sensing ability of **L2** was due to the presence of different binding sites which was substantiated by the ^1^H NMR spectra, mass spectra, and DFT studies. The chemosensor **L1** was further utilized for the selective and sensitive detection of Asp amongst all 20 amino acids. Chemosensor **L1** exhibited a ‘turn-on’ fluorescence response in the presence of Asp with a remarkable detection limit and binding constant. The ‘turn-on’ nature of the fluorescence sensing by these chemosensors allowed for the fabrication of colorimetric detection, filter-paper-based test strips, and polystyrene-film-based detection strategies.

## Data Availability

The data that support the findings of this study are available in the supplementary materials of this article.

## Supplementary Materials

The following supporting information can be downloaded at https://www.mdpi.com/article/10.3390/s23041798/s1: Figures S1–S12: FTIR, ^1^H NMR, ^13^C NMR, and HR-mass spectra of **L’**, **L1** and **L2**; Figures S13-S14: absorption spectra and emission spectra of **L1** and **L2**; Figures S15–S16: Emission spectra of **L2** and Lifetime profile of **L1** in different water percentages; Figures S17–S18: absorption spectral titrations and selectivity of **L1** and **L2**; Figure S19: Lifetime profile of **L1** in the absence and presence of Ga^3+^ ion; Figures S20–S21: ^1^H NMR spectral titration of **L1** with Al^3+^ and **L2** with Ga^3+^; Figures S22–S23: ESI^+^ mass spectrum of **L1**-Al and **L1**-Ga species; Figure S24: Job’s plot of **L1** with Al^3+^ and Ga^3+^ ions; Figure S25–S26: Contour plots and respective energy gaps of the **L1**, **L2**, **L1**–Al^3+^, **L1**–Ga^3+^, and **L2**–Ga^3+^ FMOs; Figure S27: Emission spectra of **L2** with amino acids; Figure S28: Lifetime profiles for **L1** in the absence and presence of Asp; Figure S29: ^1^H NMR spectral titration of **L1** with Asp; Figure S30: Optical images of **L1**-loaded filter paper test strips tested with different concentrations of Al^3+^ ion; Table S1: Crystallographic data collection and structure solution parameters for chemosensor **L1**; Table S2: Fluorescence lifetime parameters for **L1, L2, L1**@ f_w_ = 70%, 95% **L1**–Al^3+^, **L1**–Ga^3+^, **L2**–Ga^3+^, **L1**–Asp species; Table S3: A comparison of sensing performance of selected chemosensors for the detection of Al^3+^/Ga^3+^ ions; Table S4: xyz coordinates of the optimized geometries of **L1, L2, L1**–Al^3+^, **L1**–Ga^3+^, and **L2**–Ga^3+^; and Table S5: Energy (in eV) of frontier molecular orbitals for **L1, L2, L1**–Al^3+^, **L1**–Ga^3+^, and **L2**–Ga^3+^ species. References [4,9,18,20,80,81] are cited in the Supplementary Materials.
