# Supplementary material for: Discriminative ‘Turn-on’ Detection of Al3+ and Ga3+ Ions as Well as Aspartic Acid by Two Fluorescent Chemosensors
Source: Sensors (Basel). 2023 Feb 6;23(4):1798. doi: 10.3390/s23041798 (PMC9964346; doi:10.3390/s23041798)
Supplement: Supplementary file 1 [file sensors-23-01798-s001.zip › sensors-2153770-supplementary.pdf]

Article

Discriminative 'Turn-on' Detection of Al^{3+} and Ga^{3+} Ions as Well as Aspartic Acid by Two Fluorescent Chemosensors

Hina Goyal [1], Ibrahim Annan [1], Deepali Ahluwalia [1], Arijit Bag [2] and Rajeev Gupta [1,*]

[1] Department of Chemistry, University of Delhi, Delhi 110007, India
[2] Department of Applied Chemistry, Maulana Abul Kalam Azad University of Technology, Nadia 742149, India
* Correspondence: rgupta@chemistry.du.ac.in

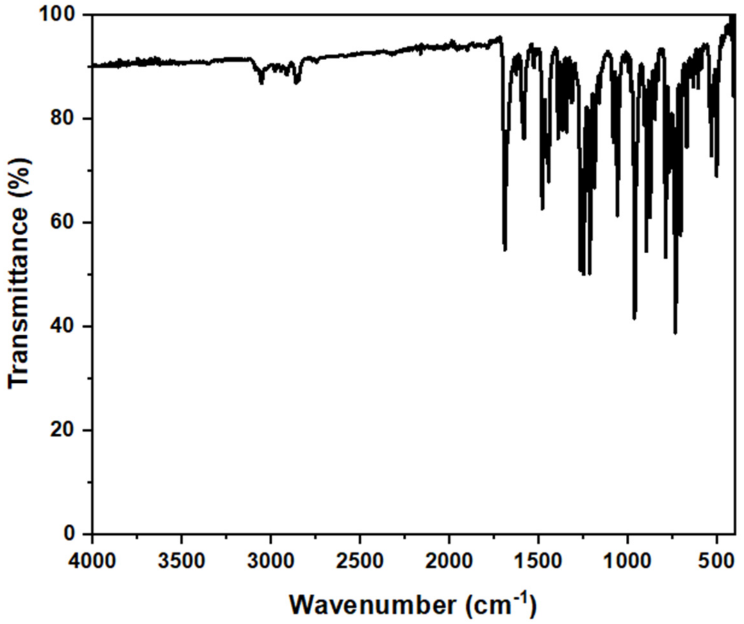

Figure S1. FTIR spectrum of **L'**.

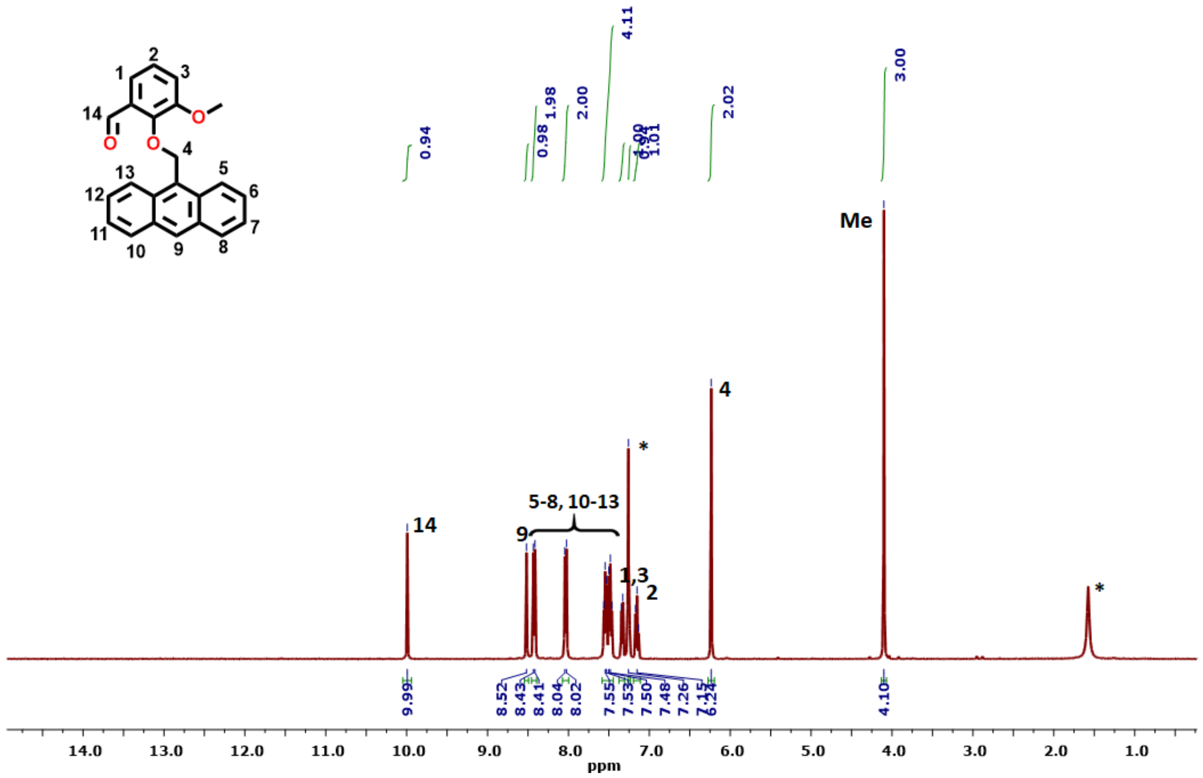

Figure S2. ^{1}H NMR spectrum of **L′** in $CDCl_3$ solvent; * represents the residual solvent and/or adventitious water peaks.

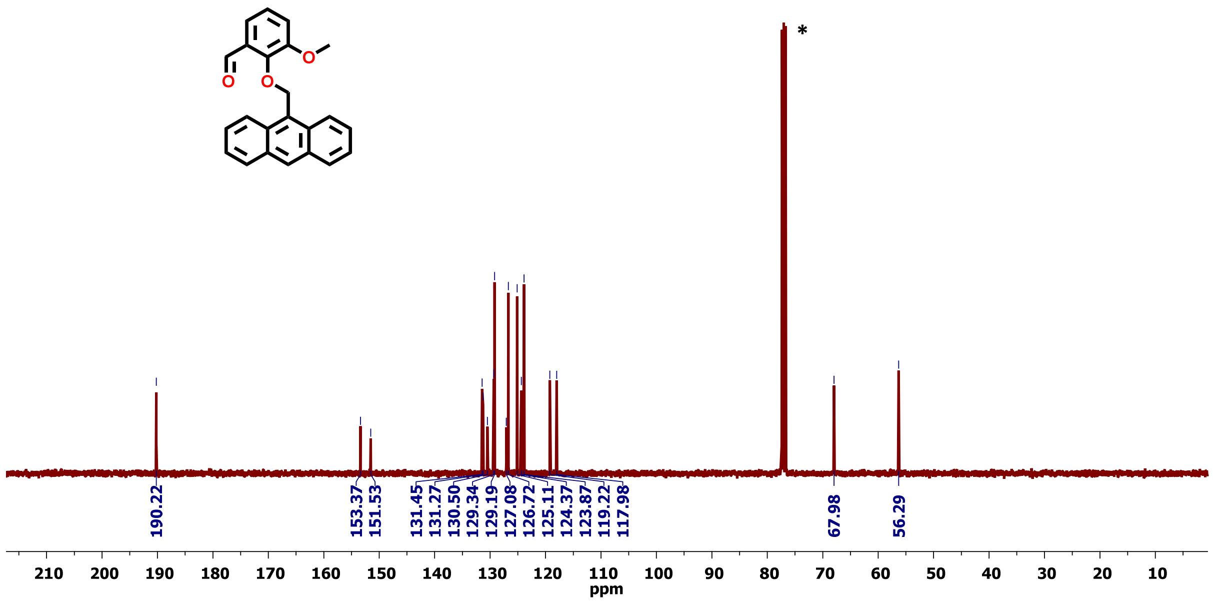

Figure S3. ^{13}C NMR spectrum of **L′** in $CDCl_3$ solvent; * represents the residual solvent and/or adventitious water peaks.

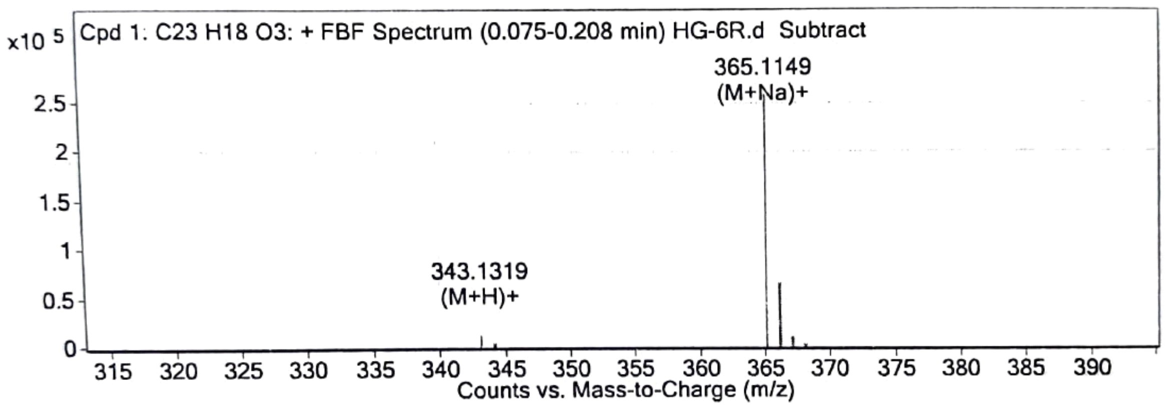

Figure S4. High-resolution ESI+ mass spectrum of **L′** in CH_3OH solvent.

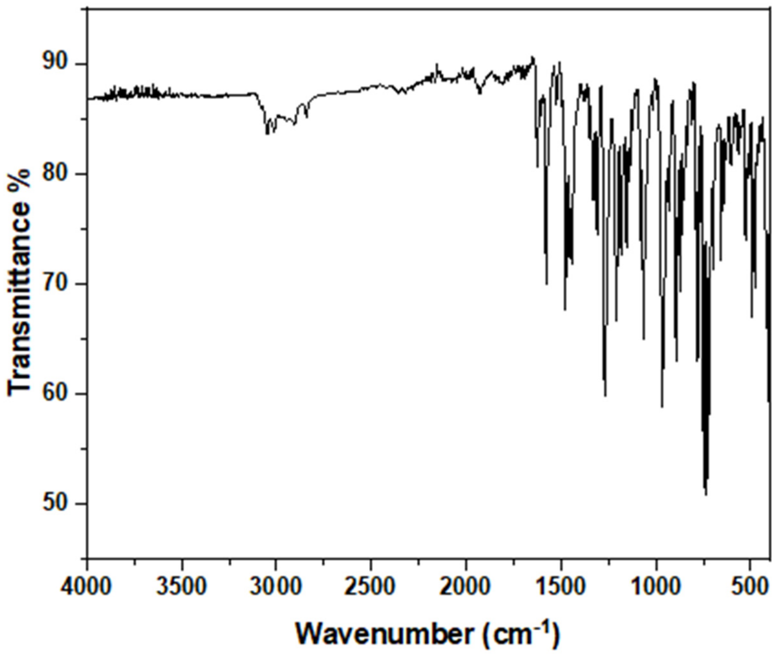

Figure S5. FTIR spectrum of **L1**.

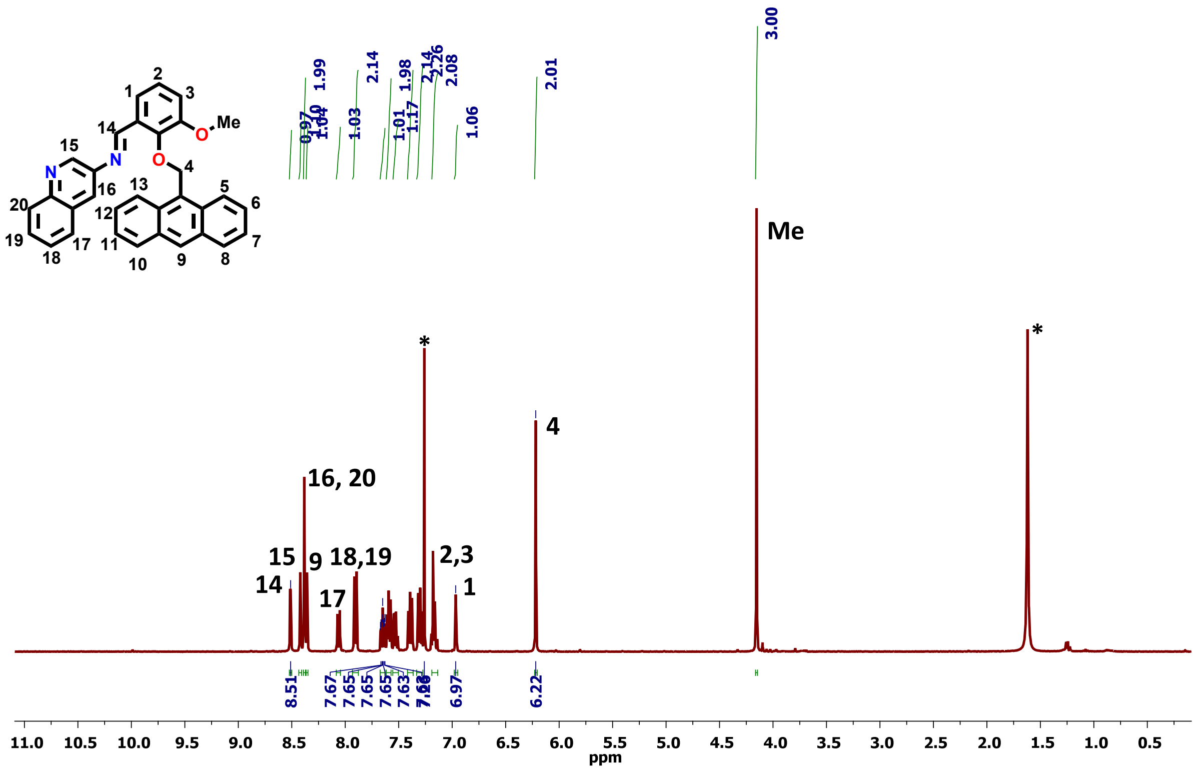

Figure S6. ^{1}H NMR spectrum of **L1** in $CDCl_3$ solvent; * represents the residual solvent and/or adventitious water peaks.

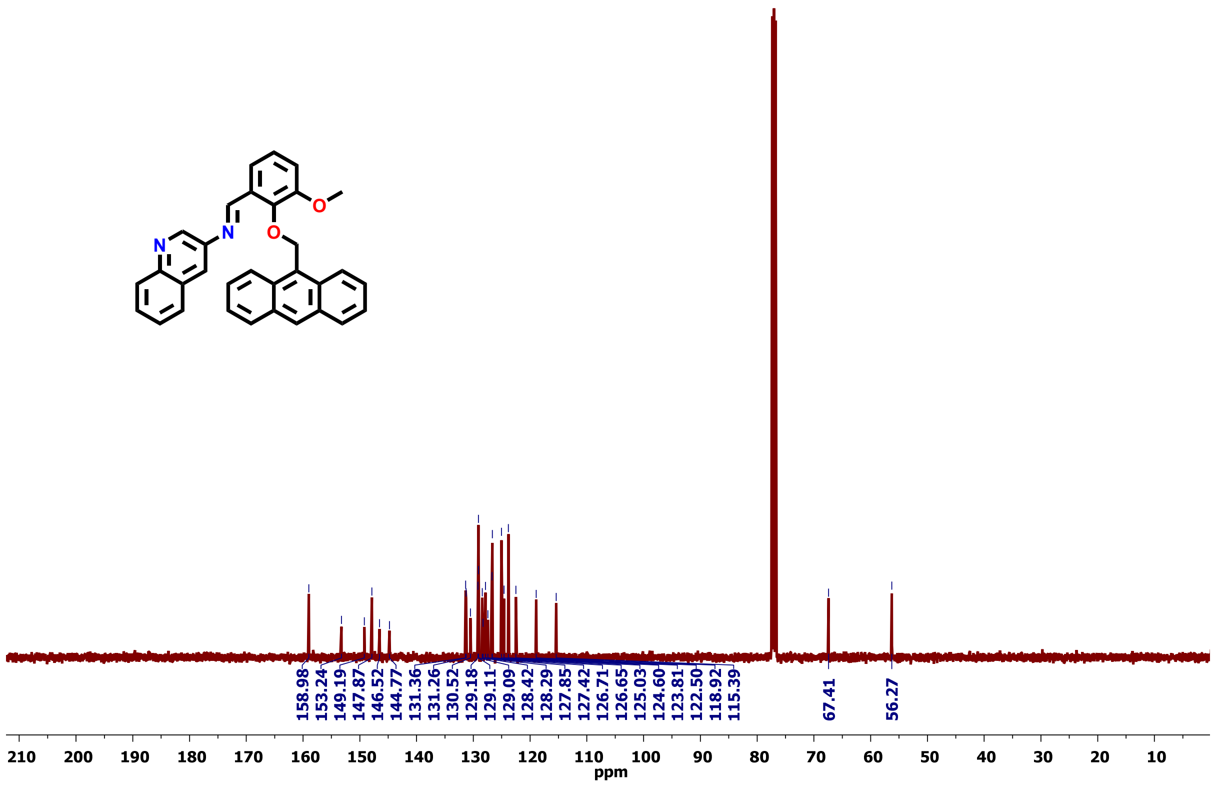

Figure S7. ^{13}C NMR spectrum of **L1** in $CDCl_3$ solvent; * represents the residual solvent and/or adventitious water peaks.

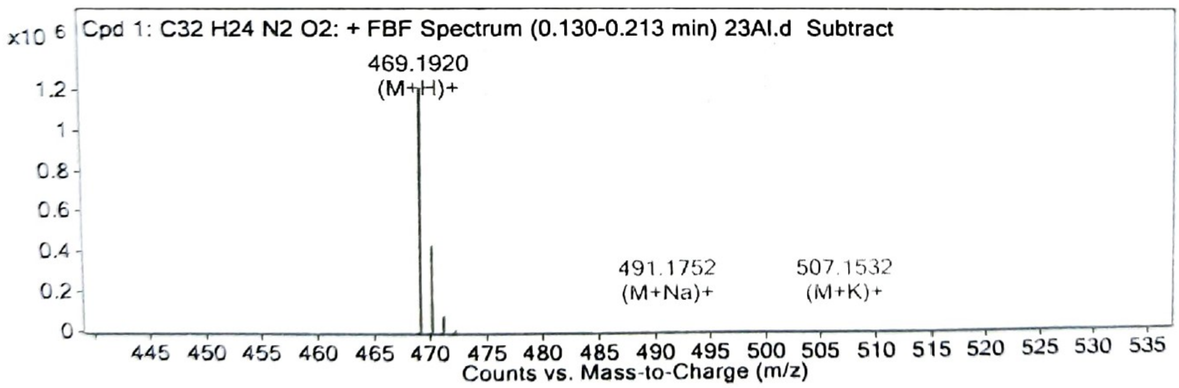

Figure S8. High-resolution ESI+ mass spectrum of **L1** in CH_3OH solvent.

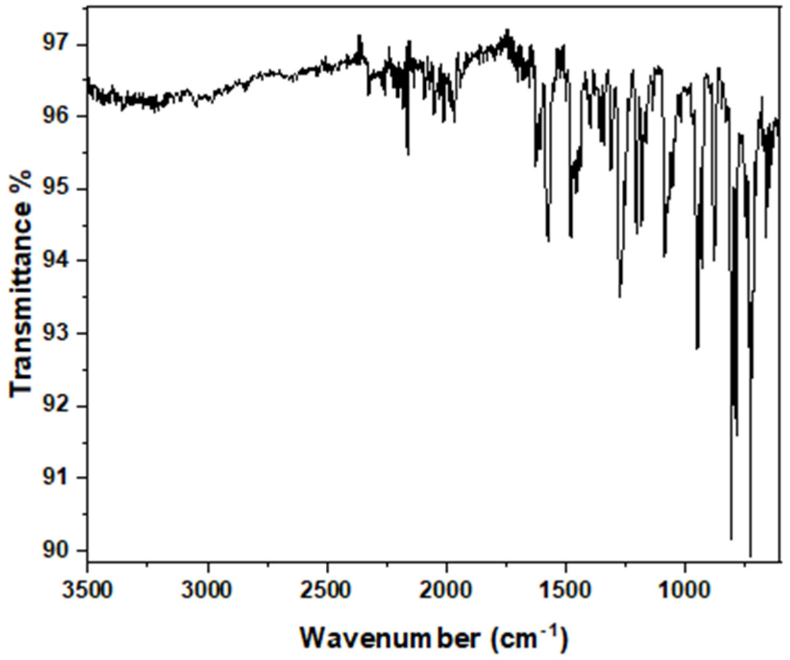

Figure S9. FTIR spectrum of **L2**.

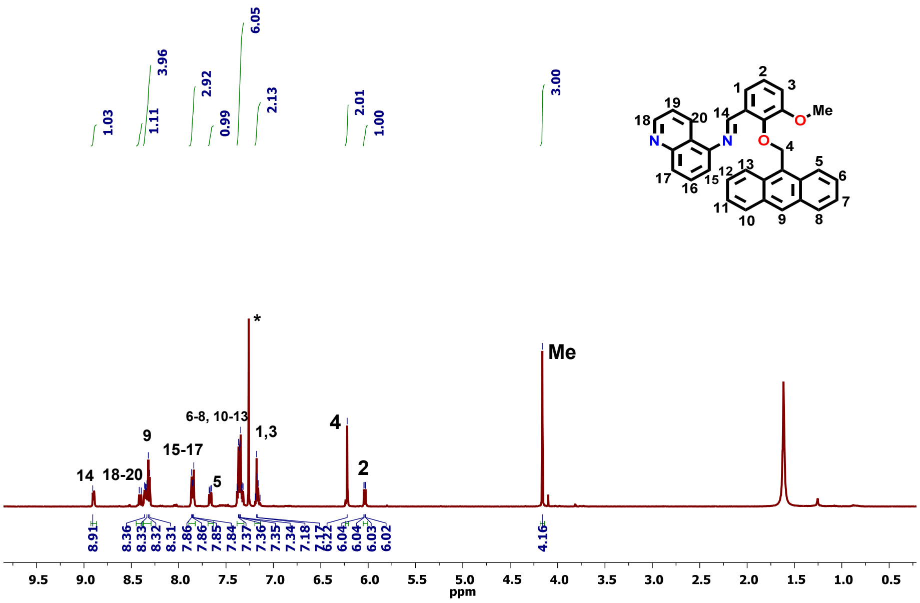

Figure S10. ^{1}H NMR spectrum of **L2** in $CDCl_3$ solvent; * represents the residual solvent and/or adventitious water peaks.

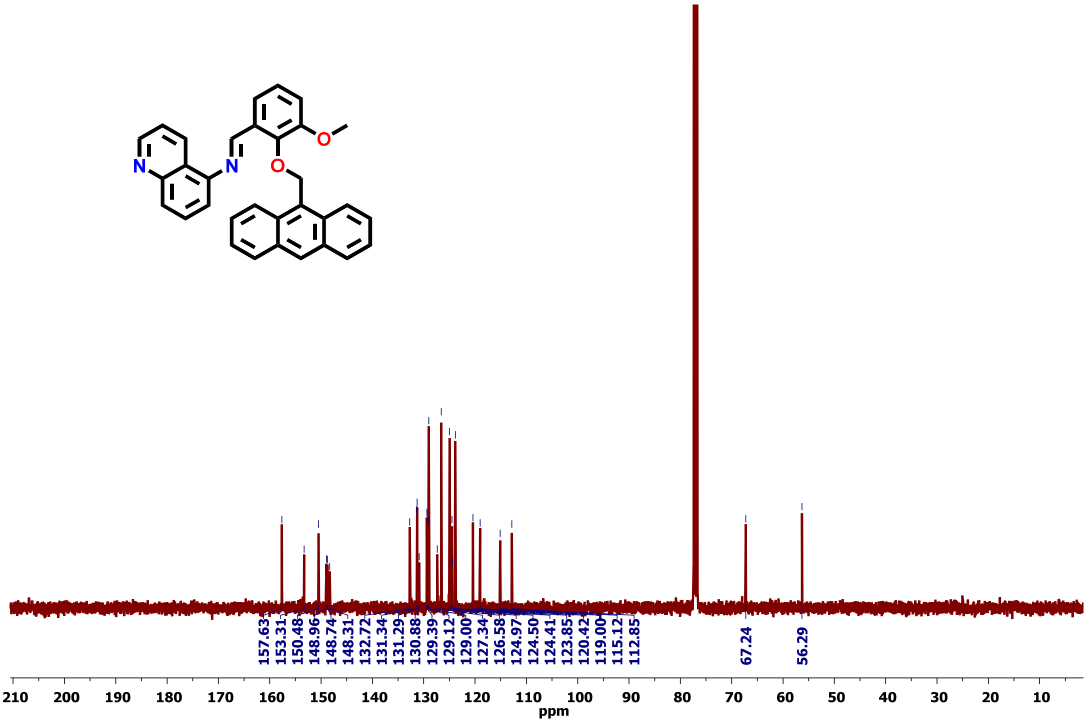

Figure S11. ^{13}C NMR spectrum of **L1** in $CDCl_3$ solvent; * represents the residual solvent and/or adventitious water peaks.

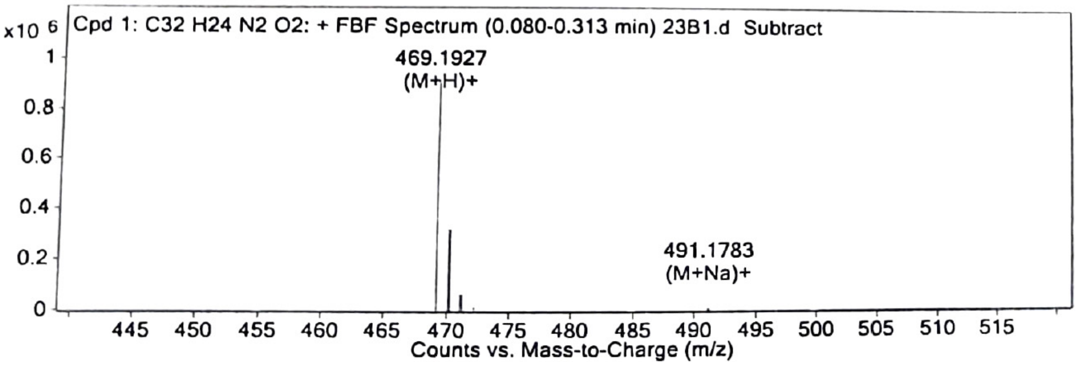

Figure S12. High-resolution ESI+ mass spectrum of **L2** in CH_3OH solvent.

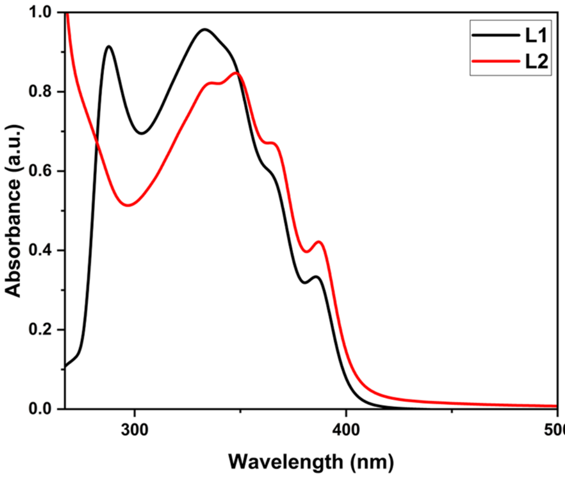

Figure S13. Absorption spectra of **L1** and **L2** (c, 20μM) in EtOH.

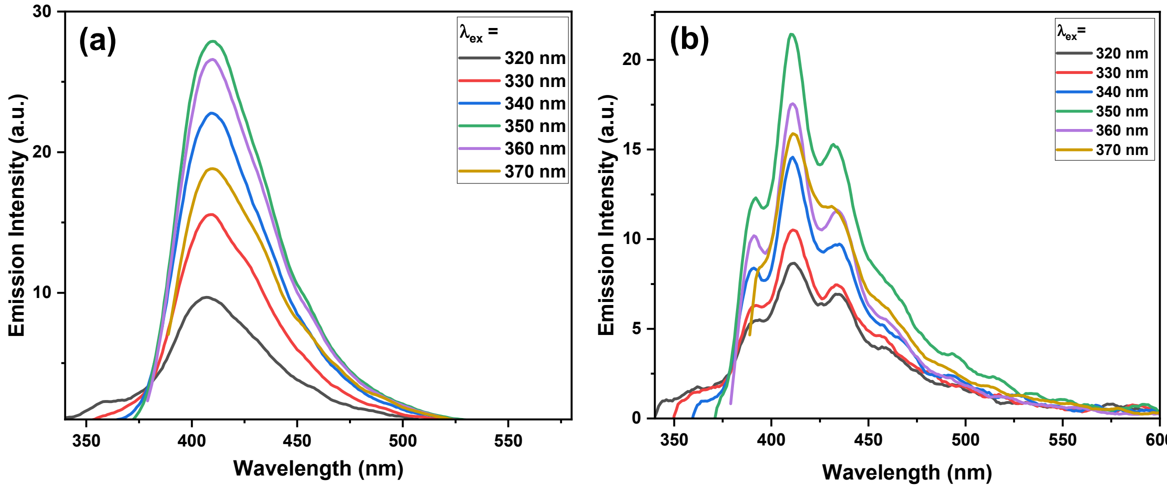

Figure S14. Emission spectra of (a) **L1** and (b) **L2** recorded in EtOH at different excitation wavelengths.

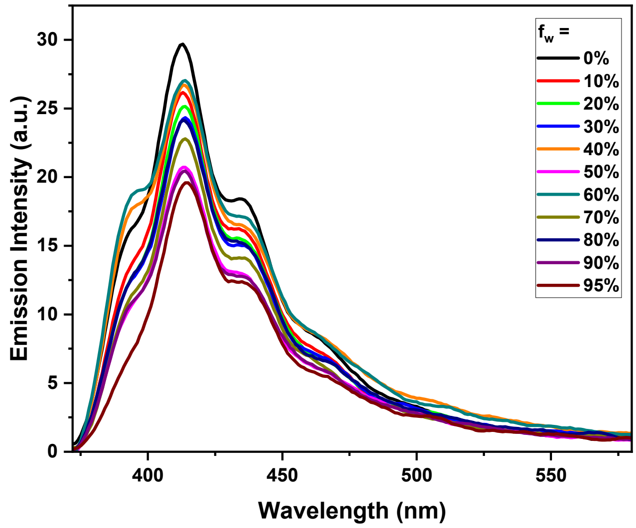

Figure S15. Emission spectra of **L2** (c, 30 μM) in MeOH–water solvent system with the fraction of water increasing from 0-95%. λ_{ex} = 350 nm.

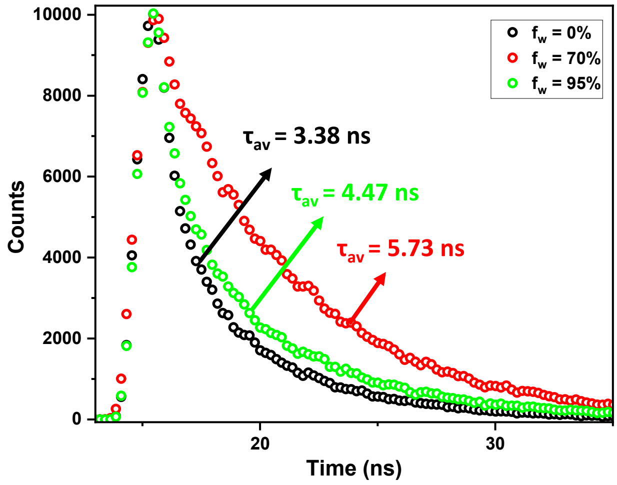

Figure S16. Lifetime profiles of **L1** in MeOH at f_w = 0%, 70%, and 95%.

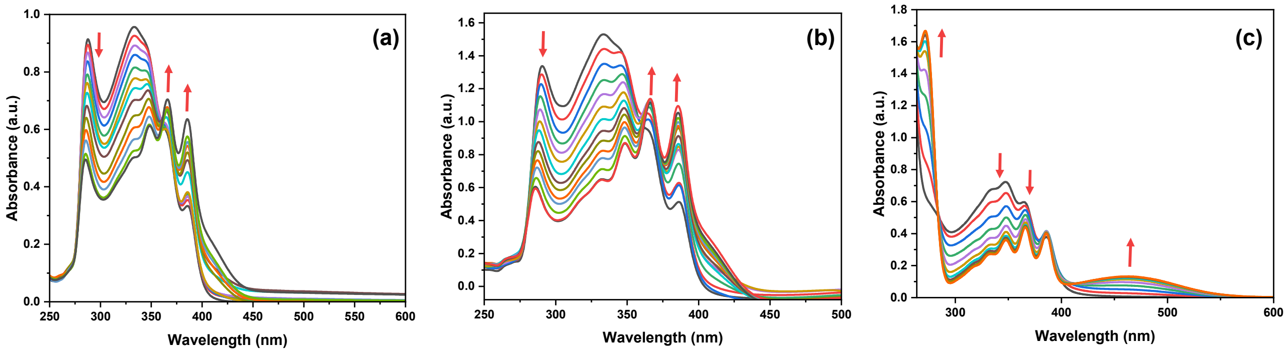

Figure S17. Absorption spectral titration of **L1** (c, 20 μM) after the addition of (a) Al^{3+} ion (0-75 μM), (b) Ga^{3+} ion (0-75 μM), and (c) **L2** (c, 20 μM) after the addition of Ga^{3+} ion (0-200 μM). All studies were conducted in EtOH.

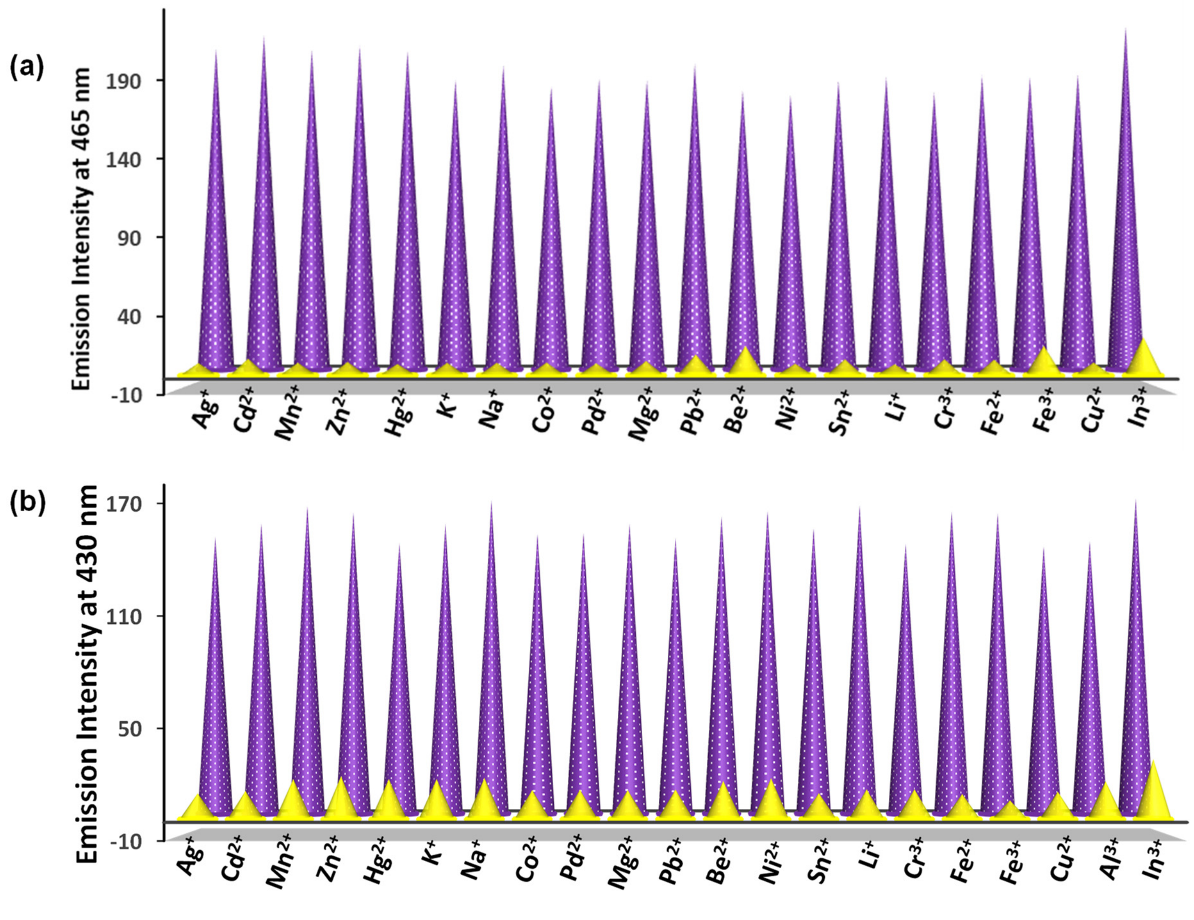

Figure S18. Selectivity of (a) chemosensor **L1** towards the Ga^{3+} ion in the presence of other metal ions: **L1** + metal ions (yellow cones) and **L1** + metal ions + Ga^{3+} ion (purple cones); (b) chemosensor **L2** towards the Ga^{3+} ion in the presence of other metal ions: **L1** + metal ions (yellow cones) and **L1** + metal ions + Ga^{3+} ion (purple cones). All studies were conducted in EtOH.

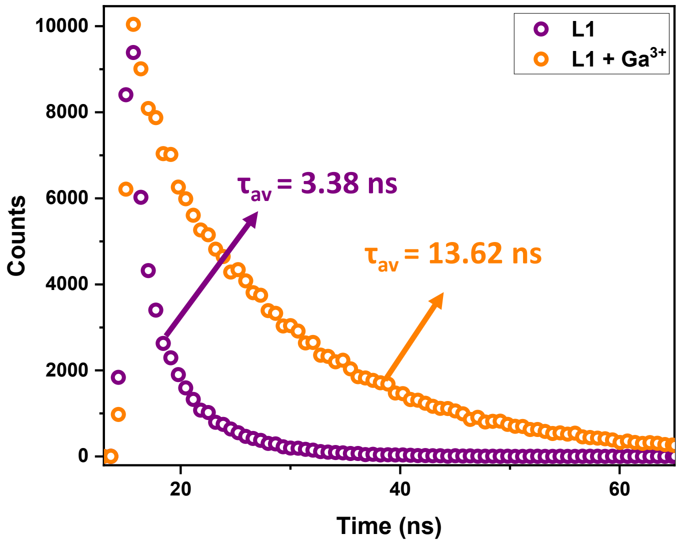

Figure S19. Lifetime profiles of **L1** in the absence and presence of Ga^{3+} ion in EtOH.

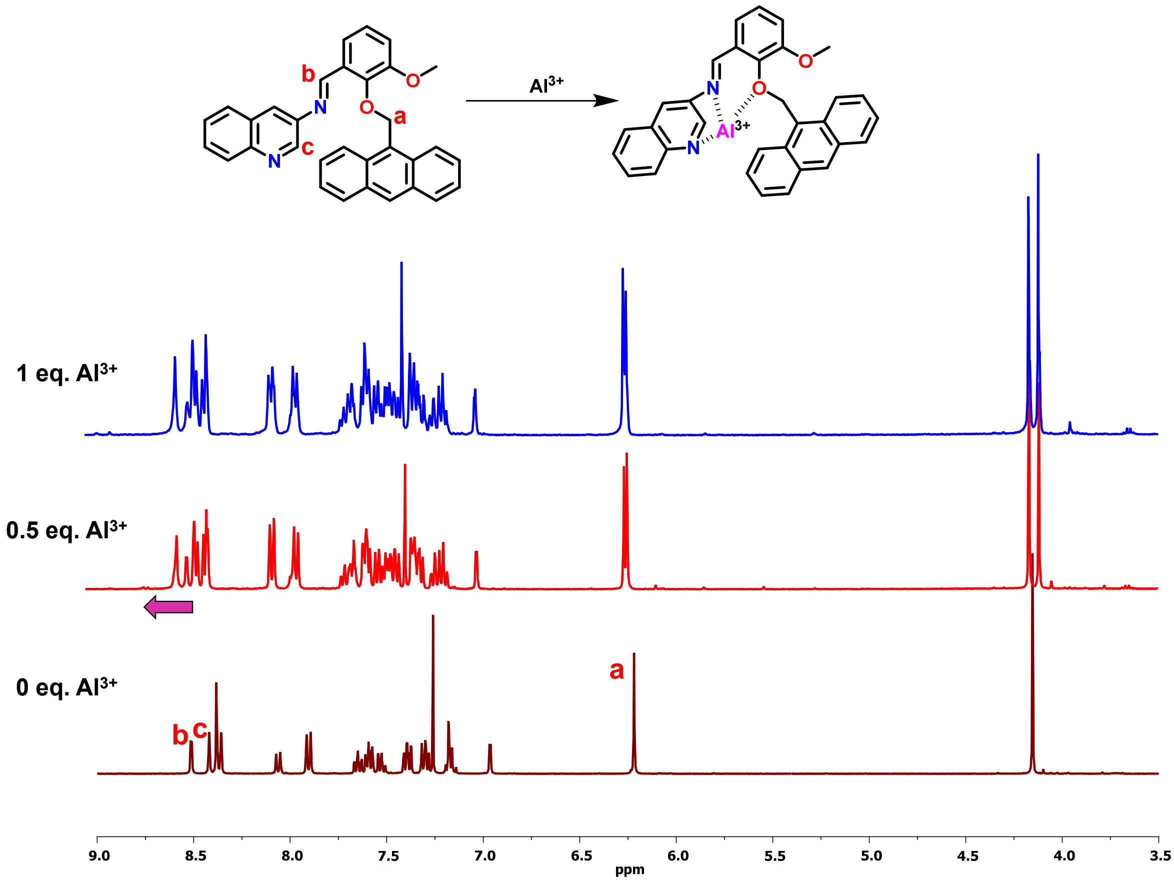

Figure S20. ^{1}H NMR spectral titration of **L1** in the presence of different amounts of Al^{3+} ion (0–1 equiv.).

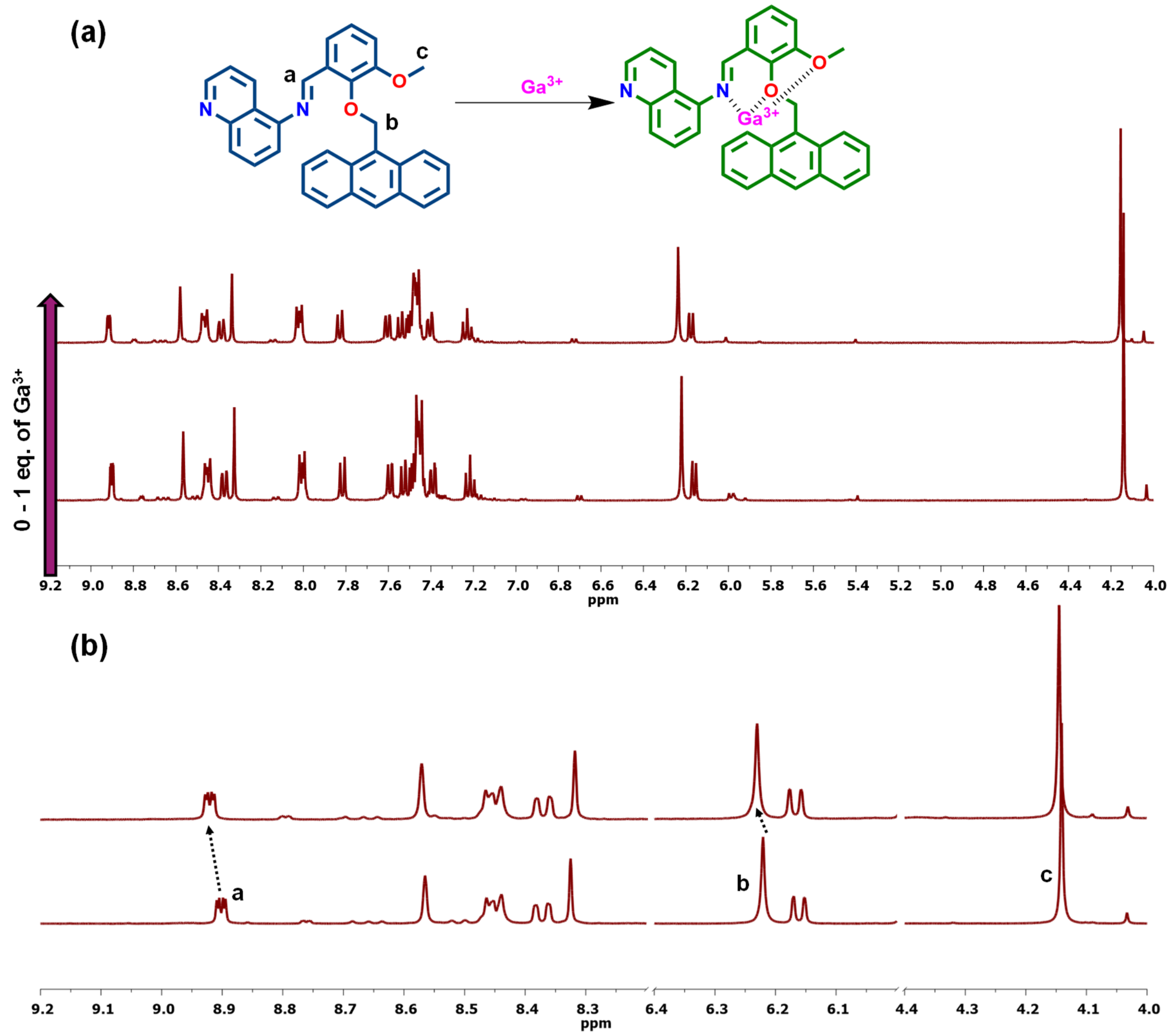

Figure S21. (a) Full-range and (b) zoomed part of the ^{1}H NMR spectra exhibiting titration of **L2** with the Ga^{3+} ion (0–1 equiv.).

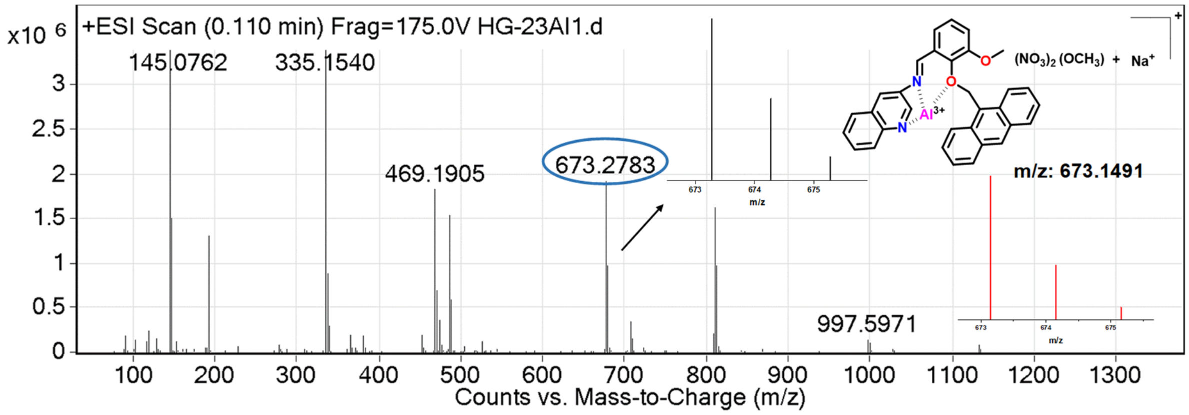

Figure S22. ESI^{+} mass spectrum of **L1**-Al^{3+} species recorded in CH_3OH along with the simulated pattern. The sources of NO_3^- and OCH_3^- ions is from aluminum precursor, $Al(NO_3)_3$, and CH_3OH solvent, respectively.

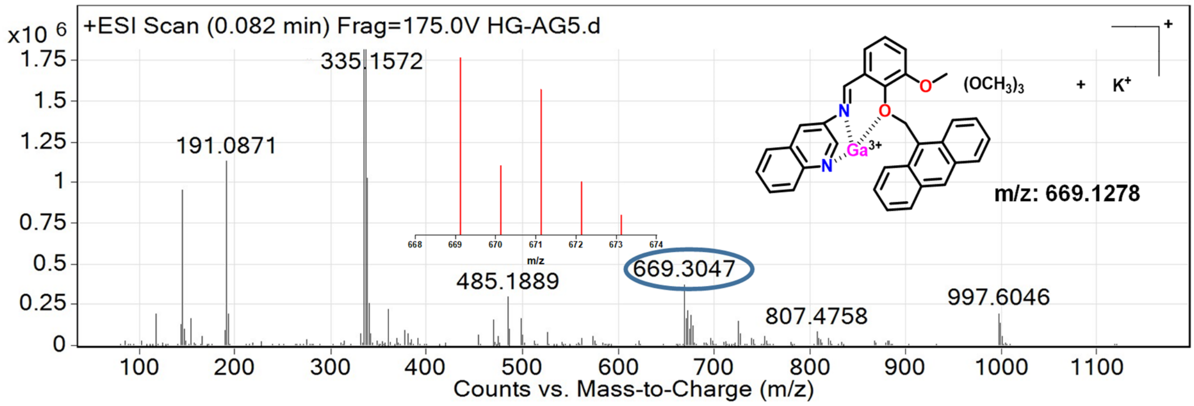

Figure S23. ESI+ mass spectrum of **L1**-Ga^{3+} species recorded in CH_3OH along with the simulated pattern. The source of OCH_3^- ion is from CH_3OH solvent.

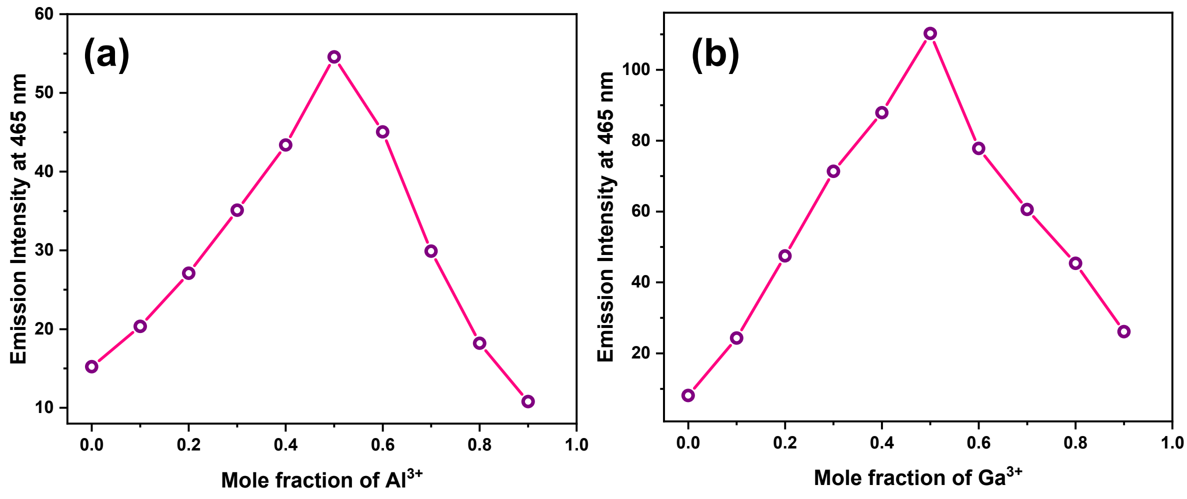

Figure S24. Job's plot for the detection of (a) Al^{3+} and (b) Ga^{3+} ions by **L1** in EtOH.

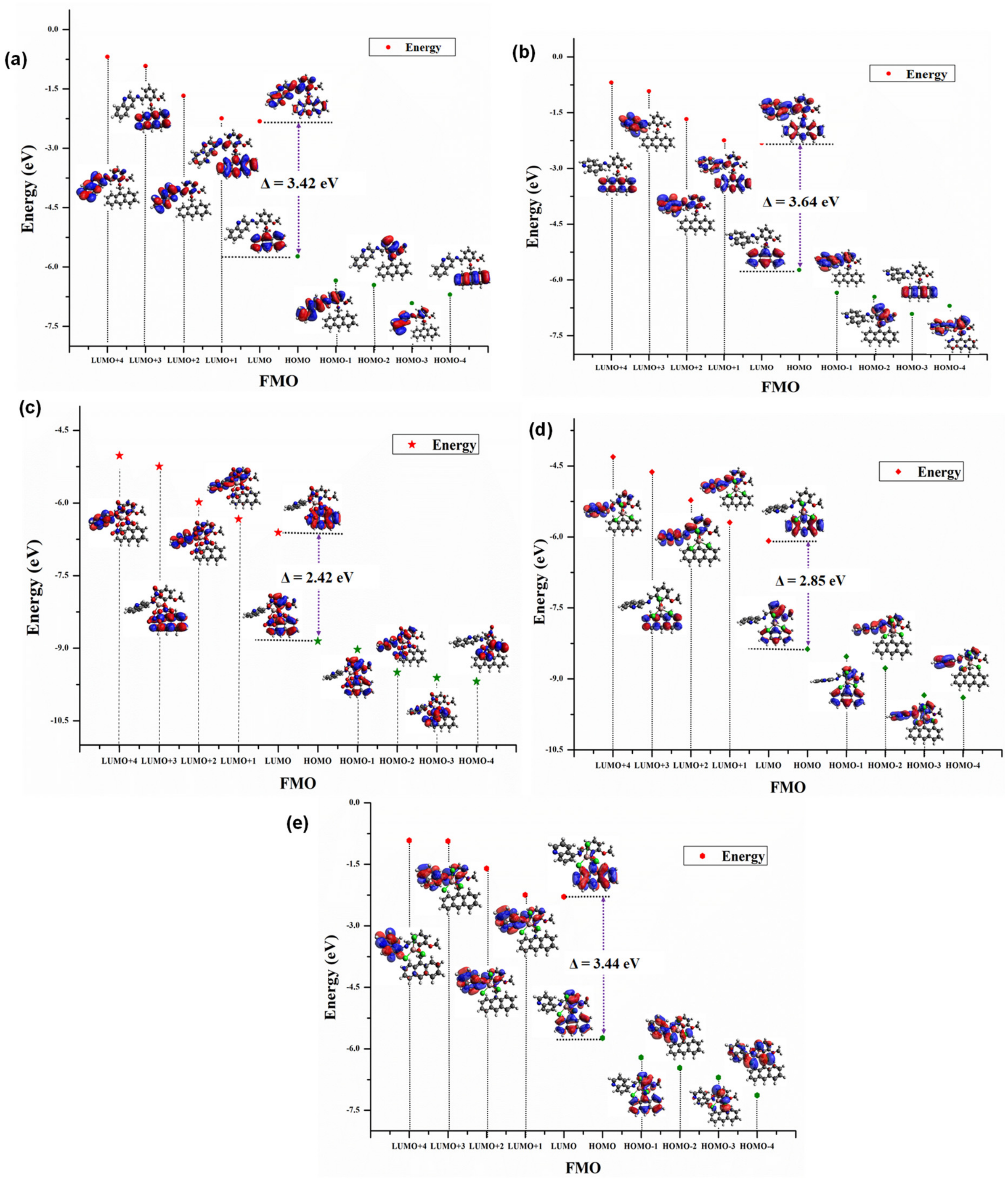

Figure S25. Contour plots of (a) **L1**, (b) **L2**, (c) **L1**–Al^{3+}, (d) **L1**–Ga^{3+}, and (e) **L2**–Ga^{3+} FMOs (HOMO-4 to LUMO+4) and their respective energy gaps calculated using the density functional theory method.

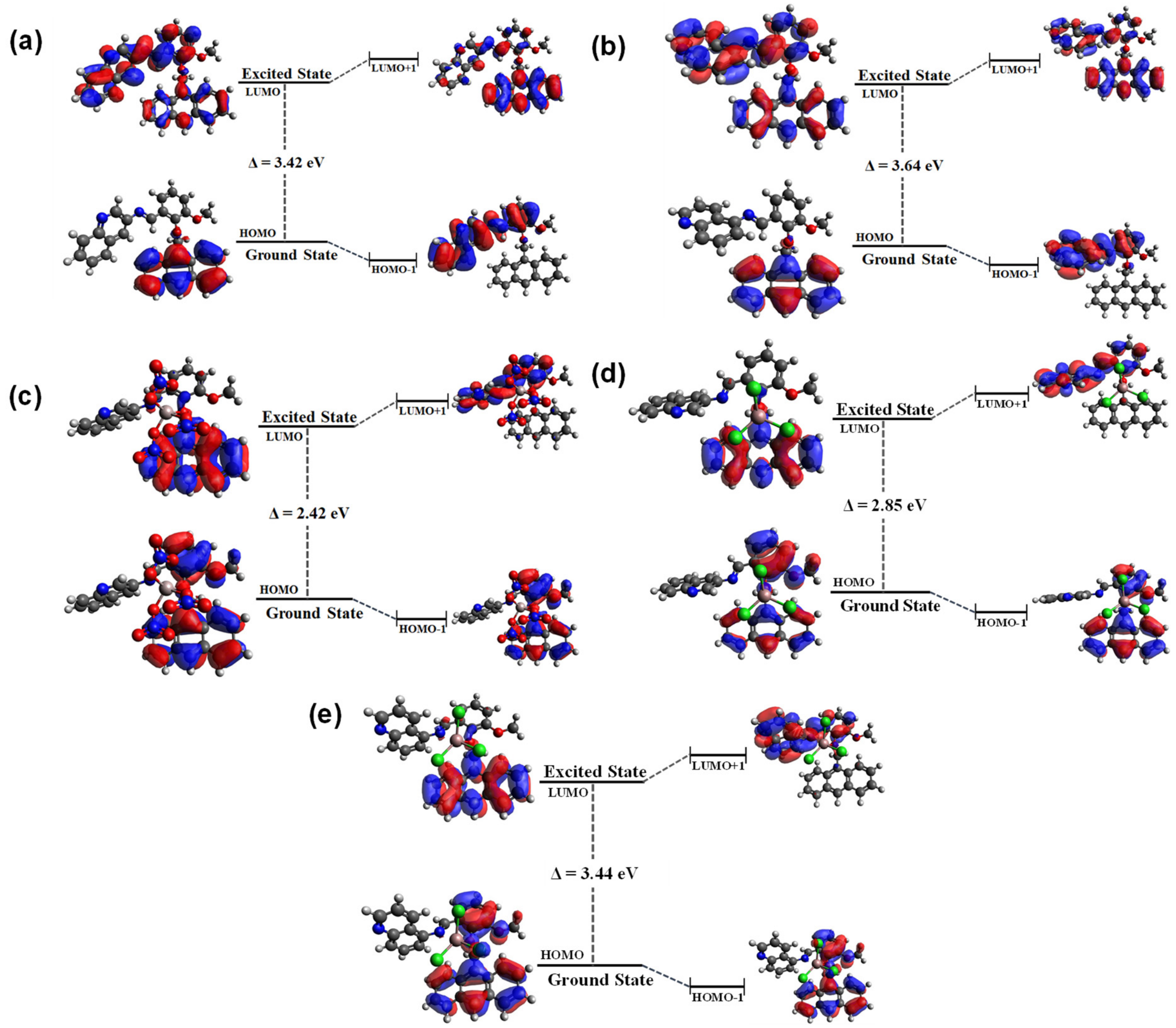

Figure S26. Contour plots of (a) **L1**, (b) **L2**, (c) **L1**–Al^{3+}, (d) **L1**–Ga^{3+}, and (e) **L2**–Ga^{3+} FMOs (HOMO-1 to LUMO+1) and their respective energy gaps calculated using the density functional theory method.

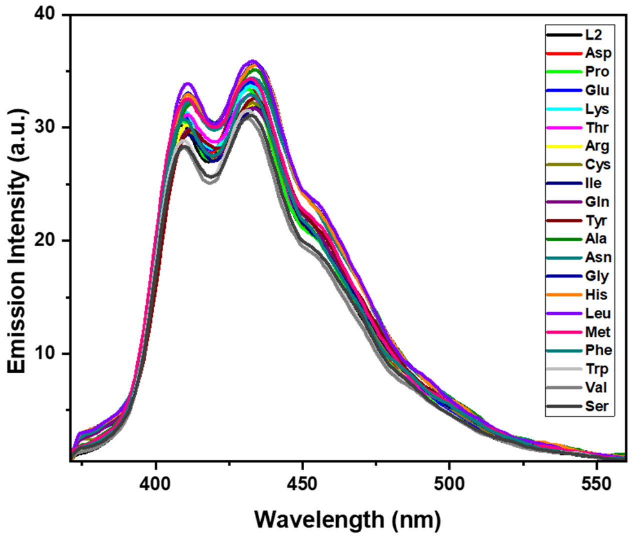

Figure S27. Emission spectra of chemosensor **L2** (c, 20 μM) in the presence of assorted amino acids.

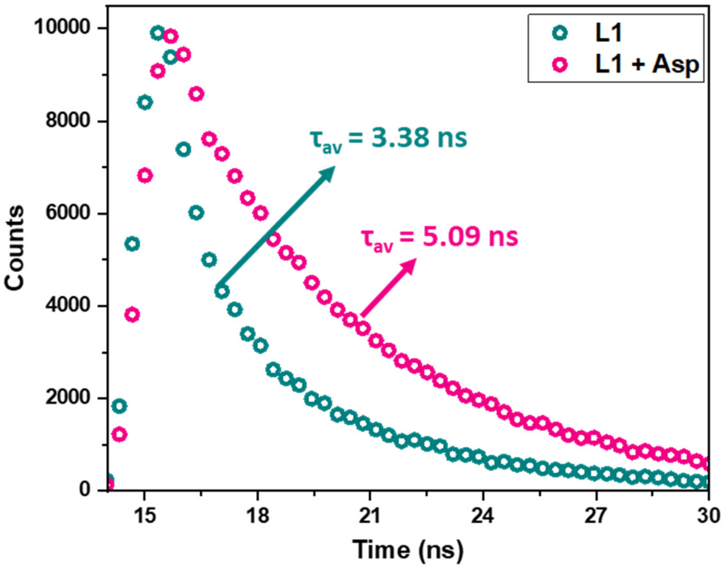

Figure S28. Lifetime profiles of **L1** in the absence and presence of Asp in EtOH.

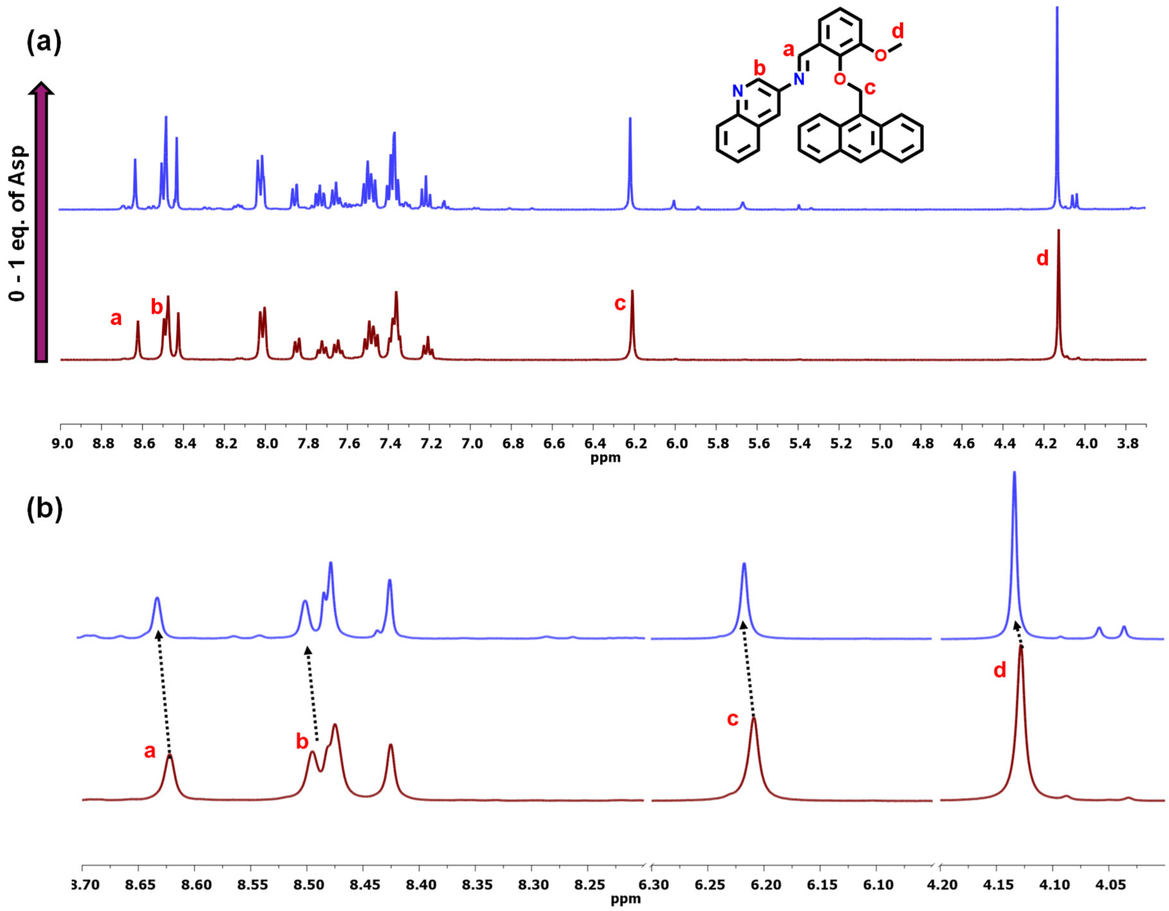

Figure S29. (a) Full-range and (b) zoomed part of the ^{1}H NMR spectra exhibiting titration of **L1** with the Asp (0–1 equiv.).

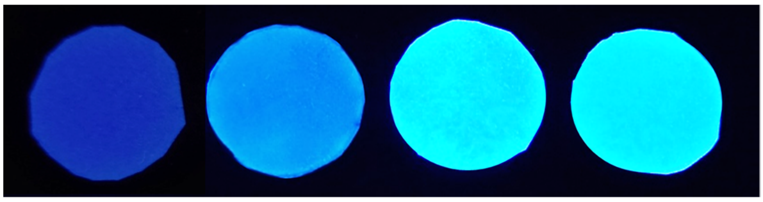

0 µM 5 µM 20 µM 50 µM

Figure S30. Optical images of **L1**-loaded filter paper test strips tested with different concentrations of Al^{3+} ion as observed under UV lamp (λ_{ex} = 365 nm).

Table S1. Crystallographic data collection and structure solution parameters for chemosensor **L1**.

Empirical formula	$C_{32}H_{24}N_2O_2$
Formula weight	468.53
Temperature/K	296
Wavelength/Å	0.71073
Crystal system	Monoclinic
Space group	*C*2/c
a/Å	50.7765 (7)
b/Å	4.6078 (6)
c/Å	21.1126 (3)
α/°	90
β/°	110.240 (8)
γ/°	90
Volume/Å^3	4634.7(11)
Z	8
ρ_{calc} g/cm^3	1.343
Absorption Coefficient/mm^{-1}	0.084
F(000)	1968.0
Crystal size/mm^3	0.21 x 0.19 x 0.18
Theta range for data collection	1.934 to 24.938°
Index ranges	-60<=h<=60, -5<=k<=5, -25<=l<=25
Reflections collected	45491
Independent reflections	4056 [R(int) = 0.0542]
Refinement method	Full–matrix least–squares on F^2
Goodness-of-fit on F^2	1.033
Final *R* indices (I>2σ (I))	R_1 = 0.0367, wR_2 = 0.0980
R indices (all data)	R_1 = 0.0450, wR_2 = 0.1052
Largest diff. peak/hole /e.Å^{-3}	0.201 and -0.220
CCDC No.	2232788

$^aR_1 = \Sigma||F_o| - |F_c||/\Sigma|F_o|$; $^b wR_2 = \{\Sigma[w(|F_o|^2 - |F_c|^2)^2]/\Sigma[wF_o^4]\}^{1/2.}$

Table S2. Fluorescence lifetime parameters for **L1, L2, L1**@ f_w = 70% and 95%, **L1**–Al^{3+}, **L1**–Ga^{3+}, **L2**–Ga^{3+}, and **L1**–Asp species.

	τ_1 (ns)	τ_2 (ns)	τ_3 (ns)	B1	B2	B3	τ_{av} (ns)
**L1**	0.729	4.681	–	0.142	0.045	–	3.38
**L2**	1.451	0.204	5.363	0.025	0.642	0.002	0.77
**L1@ f_w = 70%**	0.400	5.927	–	0.052	0.098	–	5.73
**L1@ f_w = 95%**	0.632	7.140	–	0.102	0.013	–	4.47
**L1–Al**	1.134	14.624	–	0.004	0.007	–	14.12
**L1–Ga**	1.245	14.159	–	0.004	0.007	–	13.62
**L2–Ga**	0.327	1.483	4.676	0.284	0.059	0.003	1.17
**L1–Asp**	0.738	5.326	–	0.003	0.009	–	5.09

Table S3. A comparison of the sensing performance of selected chemosensors for the detection of Al^{3+}/Ga^{3+} ions.

Chemosensor	Metal ion detected	Detection Limit	Linear range of metal ions	Reference

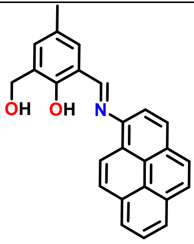	Al^{3+}, Ga^{3+}	0.38 µM, 1.17 µM	0-10 equiv.	[1]
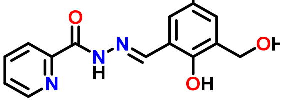	Al^{3+}, Ga^{3+}	0.06 µM, 0.20 µM	0-5 equiv.	[2]
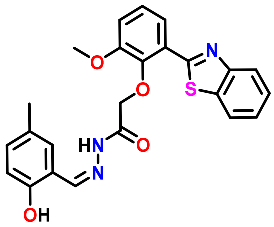	Al^{3+}, Ga^{3+}	0.09 µM, 0.03 µM	0-2 equiv. (for Al^{3+}),<br>2-4 equiv. (for Ga^{3+})	[3]
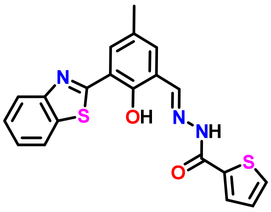	Al^{3+}	2.20 µM	0-10 equiv.	[4]
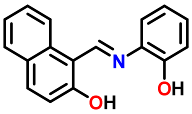	Al^{3+}	0.10 µM	0-1 equiv.	[5]
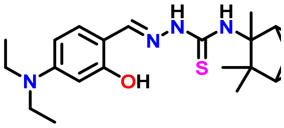	Ga^{3+}	1.18 µM	0-7.5 equiv.	[6]
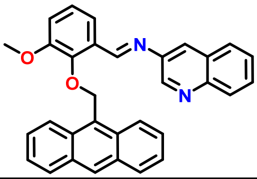	Al^{3+}, Ga^{3+}	0.19 µM, 0.22 µM	0-4 equiv.	**Present Study**

Table S4. Cartesian coordinates for the optimized geometries.

(i) L1: B3LYP/6-311+G(d,p) level optimization.

C	4.999248000	-1.498359000	1.863750000				
C	4.088444000	-0.983286000	0.979901000				
C	3.050765000	-1.799827000	0.422525000				
C	2.994357000	-3.180730000	0.836398000				
C	3.964456000	-3.678384000	1.761731000				
C	4.941498000	-2.863656000	2.262056000				
C	2.100725000	-1.302372000	-0.500761000				
C	1.994067000	-4.008684000	0.322535000	C	-4.837846000	-1.311329000	-0.560784000
C	1.040195000	-3.531806000	-0.578083000	H	-2.625619000	0.253698000	-0.923687000
C	1.081578000	-2.148184000	-0.998889000	C	-6.011319000	-1.965664000	-0.266056000
C	0.066700000	-1.716287000	-1.915791000	C	-6.939232000	-1.399655000	0.641159000
H	0.044700000	-0.688587000	-2.251235000	H	-4.123963000	-1.743086000	-1.253741000
C	-0.893076000	-2.574780000	-2.380179000	H	-6.231121000	-2.920651000	-0.729378000
C	-0.919412000	-3.936382000	-1.967091000	H	-7.859897000	-1.927308000	0.862515000
C	0.021432000	-4.396079000	-1.089701000	N	-5.257086000	1.719312000	1.556702000
H	5.774081000	-0.858484000	2.271202000				
H	4.145760000	0.062908000	0.710197000				
H	3.906283000	-4.720216000	2.058598000				
H	5.672649000	-3.249597000	2.963180000				
H	1.955967000	-5.048584000	0.631580000				
H	-1.645723000	-2.213620000	-3.071955000				
H	-1.686885000	-4.599655000	-2.349082000				

H	0.013718000	-5.430117000	-0.761597000
C	2.206322000	0.132666000	-0.944434000
H	3.240886000	0.401010000	-1.155167000
H	1.621783000	0.330421000	-1.841164000
O	1.704754000	0.993877000	0.126040000
C	1.619062000	2.333833000	-0.158463000
C	0.358780000	2.956817000	-0.156413000
C	2.785904000	3.104572000	-0.369121000
C	0.269866000	4.341463000	-0.394243000
C	2.672369000	4.471076000	-0.618164000
C	1.412661000	5.081658000	-0.632449000
H	-0.706914000	4.807202000	-0.389563000
H	3.556256000	5.070490000	-0.787692000
H	1.343769000	6.145954000	-0.824250000
O	3.967697000	2.432950000	-0.287520000
C	5.184553000	3.161148000	-0.481630000
H	5.226693000	3.597199000	-1.483355000
H	5.295934000	3.946880000	0.270534000
H	5.983705000	2.431878000	-0.367808000
C	-0.840481000	2.156650000	0.111600000
H	-0.683704000	1.084494000	0.248653000
N	-2.005498000	2.682694000	0.209382000
C	-4.144628000	2.356354000	1.278697000
C	-5.478403000	0.509199000	0.956891000
C	-4.538659000	-0.061773000	0.044015000
C	-3.344374000	0.652244000	-0.215914000
C	-3.124568000	1.862923000	0.410497000
H	-7.380799000	0.260979000	1.933325000
H	-3.988026000	3.321955000	1.753445000
C	-6.679492000	-0.187336000	1.239028000

(ii) L2: B3LYP/6-311+G(d,p) level optimization.

C	-1.122573000	3.814426000	0.108720000
C	-0.556042000	2.529821000	0.208627000
C	0.829418000	2.369108000	0.035250000
C	1.643425000	3.495077000	-0.226062000
C	1.058944000	4.755837000	-0.334646000
C	-0.322644000	4.907750000	-0.166981000
O	1.389057000	1.126530000	0.202905000
O	2.978975000	3.253309000	-0.336219000
C	3.854336000	4.354358000	-0.601130000
C	1.960151000	0.500284000	-0.988546000
C	2.438094000	-0.879367000	-0.619253000
C	-1.384969000	1.355021000	0.498702000
N	-2.659572000	1.424192000	0.609524000
C	-3.387847000	0.280069000	0.965735000
C	-4.632335000	0.041289000	0.293390000
C	-5.418407000	-1.096699000	0.654604000
C	-4.968162000	-1.955964000	1.689775000
C	-3.784908000	-1.690233000	2.337431000
C	-2.993470000	-0.576756000	1.980643000
C	1.655273000	-2.021565000	-0.911485000
C	2.155094000	-3.332896000	-0.560888000
C	3.394466000	-3.452996000	0.070065000
C	4.168693000	-2.333950000	0.382696000
C	3.682717000	-1.018963000	0.039846000
C	5.432189000	-2.469132000	1.038946000
C	6.182878000	-1.370849000	1.352774000
C	5.705063000	-0.069995000	1.028325000
C	4.501367000	0.101920000	0.397827000
C	0.369603000	-1.963118000	-1.544419000
C	-0.352094000	-3.095813000	-1.809418000
C	0.152149000	-4.381700000	-1.466969000
C	1.371296000	-4.491460000	-0.860174000
C	-5.123648000	0.879004000	-0.736035000
C	-6.317916000	0.572730000	-1.342064000
C	-7.019199000	-0.579490000	-0.919614000
N	-6.602073000	-1.386976000	0.034976000
H	-2.189993000	3.923868000	0.249803000
H	1.669312000	5.625763000	-0.535143000
H	-0.758510000	5.896503000	-0.248271000
H	4.854389000	3.928939000	-0.649792000
H	3.611231000	4.828890000	-1.555627000
H	3.809816000	5.093576000	0.203188000
H	1.193428000	0.475675000	-1.761968000
H	2.779862000	1.118305000	-1.352501000
H	-1.322485000	-3.013798000	-2.285849000
H	-0.434626000	-5.265811000	-1.687881000
H	1.769399000	-5.463738000	-0.589845000
H	-4.552817000	1.751651000	-1.027283000
H	-6.726920000	1.193651000	-2.129626000
H	-7.963510000	-0.836001000	-1.393205000

H	-0.854349000	0.406395000	0.604079000
H	-5.581436000	-2.808731000	1.954881000
H	-3.448339000	-2.338097000	3.138918000
H	-2.080554000	-0.373775000	2.527910000
H	3.763100000	-4.440974000	0.327668000
H	5.780918000	-3.466715000	1.284236000
H	7.138791000	-1.483209000	1.851380000
H	6.302863000	0.796040000	1.289761000
H	4.156193000	1.104576000	0.183776000
H	-0.054160000	-1.006691000	-1.817621000

(iii) L1-Al^{3+} complex: optimized at B3LYP functional and LANL2DZ basis set for Al^{3+} ion and 6311+G(d,p) for C, O, N, and H.

C	-1.553221000	-3.063623000	-2.354567000
C	-0.591431000	-2.117512000	-2.118050000
C	0.652668000	-2.451468000	-1.495038000
C	0.849101000	-3.835095000	-1.130514000
C	-0.180102000	-4.792456000	-1.391114000
C	-1.352395000	-4.422051000	-1.987064000
C	1.688236000	-1.514275000	-1.238059000
C	2.049858000	-4.227598000	-0.537491000
C	3.076502000	-3.319007000	-0.277457000
C	2.899539000	-1.932703000	-0.629400000
C	3.979028000	-1.039820000	-0.346651000
H	3.873688000	0.013811000	-0.562934000
C	5.141951000	-1.489239000	0.222129000
C	5.312452000	-2.859405000	0.561522000
C	4.302163000	-3.747682000	0.319390000
H	-2.483935000	-2.772114000	-2.827746000
H	-0.794542000	-1.097252000	-2.411079000
H	-0.005444000	-5.823900000	-1.104612000
H	-2.127962000	-5.153283000	-2.182091000
H	2.189733000	-5.271373000	-0.274222000
H	5.943228000	-0.788068000	0.426730000
H	6.240297000	-3.190327000	1.013339000
H	4.411660000	-4.796081000	0.574917000
C	1.588859000	-0.134870000	-1.781916000
H	2.544196000	0.224681000	-2.145395000
H	0.841376000	-0.038364000	-2.560768000
O	1.183057000	0.982642000	-0.764265000
C	1.167804000	2.228425000	-1.415611000
C	-0.062218000	2.844688000	-1.678891000
C	2.373161000	2.831823000	-1.801794000
C	-0.092758000	4.049610000	-2.399856000
C	2.318681000	4.046012000	-2.499337000
C	1.095509000	4.640125000	-2.801285000
H	-1.044884000	4.518329000	-2.615037000
H	3.234494000	4.529837000	-2.808823000
H	1.080612000	5.574293000	-3.348349000
O	3.513976000	2.191503000	-1.464664000
C	4.775534000	2.771770000	-1.838237000
H	4.858506000	2.854150000	-2.924032000
H	4.903339000	3.750636000	-1.371431000
H	5.530312000	2.085270000	-1.463047000
C	-1.317667000	2.315206000	-1.185354000
H	-2.215879000	2.659359000	-1.694540000
N	-1.444586000	1.509130000	-0.176977000
C	-3.345011000	1.070437000	1.361727000
C	-5.312271000	0.108642000	0.638339000
C	-4.820972000	-0.006200000	-0.697152000
C	-3.512894000	0.463430000	-0.960049000
C	-2.771397000	1.008699000	0.061448000
H	-6.976812000	-0.260919000	1.953433000
H	-2.773604000	1.498074000	2.176407000
C	-6.617769000	-0.354645000	0.935381000

C	-5.647767000	-0.583745000	-1.695334000
H	-3.102174000	0.366415000	-1.958701000
C	-6.911193000	-1.023931000	-1.377532000
C	-7.398503000	-0.908322000	-0.053150000
H	-5.269702000	-0.669445000	-2.708088000
H	-7.541144000	-1.463126000	-2.142152000
H	-8.396494000	-1.261145000	0.179803000
N	-4.556788000	0.650412000	1.639964000
Al	0.199095000	0.956553000	0.959421000
O	-0.658374000	-0.655406000	1.111936000
O	0.609190000	2.952558000	1.172649000
N	-0.076427000	3.010774000	2.257170000
O	-0.218475000	4.002542000	2.914742000
O	-0.596432000	1.868308000	2.553648000
N	2.061733000	0.198464000	3.027752000
O	1.842962000	0.612785000	1.786669000
O	3.232321000	0.126805000	3.372602000
O	1.104250000	-0.081460000	3.739021000
N	-0.413060000	-1.786835000	1.782062000
O	-1.395149000	-2.350846000	2.238718000
O	0.732164000	-2.190631000	1.856689000

(iv) L1-Ga^{3+} complex: optimized at B3LYP functional and LANL2DZ basis set for Ga^{3+} ion and 6311+G(d,p) for C, O, N, and H.

C	-1.919877000	-3.046739000	-0.765436000	C	-6.388273000	0.170668000	-1.716419000
C	-0.943354000	-2.101669000	-0.936038000	H	-3.813849000	1.046143000	-2.003253000
C	0.443564000	-2.435259000	-0.843819000	C	-7.638114000	-0.305901000	-1.395781000
C	0.768212000	-3.810333000	-0.549966000	C	-7.928367000	-0.723398000	-0.074540000
C	-0.279466000	-4.765926000	-0.368412000	H	-6.161706000	0.491628000	-2.727521000
C	-1.591113000	-4.399533000	-0.477625000	H	-8.408005000	-0.364692000	-2.156451000

C	1.492879000	-1.496712000	-1.037369000
C	2.105898000	-4.197824000	-0.463087000
C	3.150514000	-3.295868000	-0.669795000
C	2.849363000	-1.916189000	-0.966211000
C	3.958140000	-1.042758000	-1.194580000
H	3.784875000	0.006574000	-1.382871000
C	5.248612000	-1.500032000	-1.147782000
C	5.536877000	-2.860199000	-0.851685000
C	4.511060000	-3.730980000	-0.614064000
H	-2.961746000	-2.756990000	-0.840739000
H	-1.239036000	-1.078502000	-1.114509000
H	-0.005858000	-5.791764000	-0.146806000
H	-2.380869000	-5.129422000	-0.343673000
H	2.340537000	-5.234900000	-0.244536000
H	6.066084000	-0.811119000	-1.328456000
H	6.566166000	-3.197295000	-0.814637000
H	4.708404000	-4.772740000	-0.385764000
C	1.173734000	-0.108222000	-1.478616000
H	1.939393000	0.301183000	-2.129639000
H	0.208505000	-0.034536000	-1.967544000
O	1.080518000	0.927311000	-0.340456000
C	0.976804000	2.243432000	-0.846711000
C	-0.264896000	2.842111000	-1.108971000
C	2.179927000	2.920916000	-1.107708000
C	-0.270207000	4.134732000	-1.662080000
C	2.139175000	4.212360000	-1.645296000
C	0.913850000	4.808024000	-1.923181000
H	-1.221918000	4.607596000	-1.871968000
H	3.056257000	4.746459000	-1.849911000
H	0.889411000	5.805693000	-2.344097000
O	3.317528000	2.254550000	-0.809073000
C	4.577180000	2.921976000	-0.978485000
H	4.751306000	3.165656000	-2.029114000
H	4.622004000	3.826784000	-0.368257000
H	5.327813000	2.213312000	-0.637199000
C	-1.586916000	2.271140000	-0.824351000
H	-2.414849000	2.870706000	-1.220008000
N	-1.811978000	1.215190000	-0.141044000
C	-3.529552000	0.312551000	1.308782000
C	-5.669042000	-0.169403000	0.607263000
C	-5.375162000	0.254637000	-0.725321000
C	-4.073990000	0.742318000	-0.994915000
C	-3.141983000	0.789465000	0.021283000
H	-7.170912000	-0.976602000	1.921497000
H	-2.789006000	0.321845000	2.103092000
C	-6.964802000	-0.658166000	0.906204000
H	-8.917758000	-1.097774000	0.161879000
N	-4.729279000	-0.130197000	1.601487000
Cl	3.352735000	0.210982000	2.130722000
Cl	-0.033889000	-0.948452000	2.397882000
Cl	0.688789000	2.629131000	2.431782000
Ga	1.255980000	0.654172000	1.661785000

(v) L2-Ga^{3+} complex: optimized at B3LYP functional and LANL2DZ basis set for Ga^{3+} ion and 6311+G(d,p) for C, O, N, and H.

C	0.868031000	-3.923033000	-2.104442000
C	0.621544000	-2.660271000	-1.514341000
C	-0.667534000	-2.365604000	-1.015666000
C	-1.699421000	-3.328140000	-1.064955000
C	-1.425732000	-4.593162000	-1.630978000
C	-0.152599000	-4.881091000	-2.152179000
O	-0.917088000	-1.062681000	-0.484217000
O	-2.928778000	-2.960105000	-0.545962000
C	-4.027902000	-3.940895000	-0.544503000
C	-1.917752000	-0.213531000	-1.410252000
C	-2.334139000	1.090115000	-0.819897000
C	1.729856000	-1.717142000	-1.425646000
N	1.824583000	-0.736714000	-0.555985000
C	2.970330000	0.154974000	-0.742699000
C	4.065301000	0.179486000	0.184040000
C	5.142744000	1.107842000	-0.059857000
C	5.114203000	1.953783000	-1.209751000
C	4.057224000	1.874066000	-2.104284000
C	2.983292000	0.968169000	-1.870431000
C	-1.641582000	2.291700000	-1.178002000
C	-2.111149000	3.565810000	-0.659142000
C	-3.250426000	3.602465000	0.165676000
C	-3.963843000	2.435915000	0.497984000
C	-3.505031000	1.150569000	0.002355000
C	-5.146750000	2.511435000	1.314807000
C	-5.862177000	1.372629000	1.629639000
C	-5.412557000	0.098262000	1.147770000
H	-3.939316000	-0.994349000	0.063220000
H	-0.117944000	1.405628000	-2.482232000
H	0.987634000	3.506257000	-3.047838000
H	0.186258000	5.682628000	-2.110326000
H	-1.794184000	5.719240000	-0.597086000
H	3.410106000	-1.414281000	1.528624000
H	5.399603000	-1.211686000	3.019819000
H	7.161623000	0.494289000	2.495556000
Ga	0.343693000	-0.511952000	0.988675000
Cl	1.151820000	-2.431275000	2.010456000
Cl	-1.442675000	-0.337175000	2.499173000
Cl	1.115025000	1.597210000	1.458208000

C	-4.271543000	-0.010913000	0.371579000
C	-0.498223000	2.324997000	-2.052808000
C	0.131060000	3.513745000	-2.378842000
C	-0.327948000	4.763162000	-1.845710000
C	-1.424168000	4.782066000	-1.005988000
C	4.178775000	-0.678866000	1.320703000
C	5.286453000	-0.572355000	2.150463000
C	6.292364000	0.393077000	1.850176000
N	6.236236000	1.202236000	0.786125000
H	1.854270000	-4.147010000	-2.499471000
H	-2.200547000	-5.350272000	-1.670622000
H	0.035039000	-5.855075000	-2.592206000
H	-4.868051000	-3.420393000	-0.084373000
H	-4.283721000	-4.235561000	-1.568139000
H	-3.757670000	-4.817688000	0.053512000
H	-1.347159000	-0.124740000	-2.332341000
H	-2.739368000	-0.912398000	-1.537236000
H	2.548137000	-1.871511000	-2.130162000
H	5.940062000	2.641783000	-1.361352000
H	4.033962000	2.504267000	-2.988236000
H	2.157503000	0.926673000	-2.572765000
H	-3.596714000	4.561346000	0.546674000
H	-5.466877000	3.486590000	1.674099000
H	-6.757759000	1.431277000	2.241958000
H	-5.973290000	-0.795336000	1.410297000

Table S5. Energy (in eV) of the frontier molecular orbitals for **L1, L2, L1**–Al^{3+}, **L1**–Ga^{3+}, and **L2**–Ga^{3+} species.

FMO	HOMO-4	HOMO-3	HOMO-2	HOMO-1	HOMO	LUMO	LUMO+1	LUMO+2	LUMO+3	LUMO+4
**L1**	-6.96	-6.92	-6.46	-6.35	-5.73	-2.32	-2.24	-1.67	-0.92	-0.68
**L2**	-7.13	-6.96	-6.46	-6.21	-5.73	-2.09	-2.24	-1.601	-0.93	-0.92
**L1-Al^{3+}**	-9.68	-9.61	-9.49	-9.03	-8.85	-6.61	-6.33	-5.98	-5.24	-5.02
**L1-Ga^{3+}**	-9.39	-9.34	-8.77	-8.53	-8.37	-6.08	-5.69	-5.22	-4.63	-4.03
**L2-Ga^{3+}**	-7.13	-6.96	-6.46	-6.21	-5.73	-2.29	-2.25	-1.60	-0.93	-0.92

References

1. Kumar, V.; Kumar, P.; Kumar, S.; Singhal, D.; Gupta, R. Turn-On Fluorescent Sensors for the Selective Detection of Al^{3+} (and Ga^{3+}) and PPi Ions. *Inorg. Chem.* **2019**, *58*, 10364–10376, doi:10.1021/acs.inorgchem.9b01550.
2. Mishra, S.; Mamidi, P.; Chattopadhyay, S.; Singh, A.K. Economically Viable Multi-Responsive Probes for Fluorimetric Detection of Trace Levels of Ga^{3+}, Al^{3+} and PPi in near Aqueous Medium. *J. Photochem. Photobiol. Chem.* **2023**, *434*, 114225, doi:10.1016/j.jphotochem.2022.114225.
3. Liu, Q.; Liu, Y.; Xing, Z.; Huang, Y.; Ling, L.; Mo, X. A Novel Dual-Function Probe for Fluorescent Turn-on Recognition and Differentiation of Al^{3+} and Ga^{3+} and Its Application. *Spectrochim. Acta. A. Mol. Biomol. Spectrosc.* **2023**, *287*, 122076, doi:10.1016/j.saa.2022.122076.
4. Chen, Y.; Wei, T.; Zhang, Z.; Chen, T.; Li, J.; Qiang, J.; Lv, J.; Wang, F.; Chen, X. A Benzothiazole-Based Fluorescent Probe for Ratiometric Detection of Al^{3+} in Aqueous Medium and Living Cells. *Ind. Eng. Chem. Res.* **2017**, *56*, 12267–12275, doi:10.1021/acs.iecr.7b02979.

5. Guo, A.; Zhu, R.; Ren, Y.; Dong, J.; Feng, L. A “Turn-on” Fluorescent Chemosensor for Aluminum Ion and Cell Imaging Application. *Spectrochim. Acta. A. Mol. Biomol. Spectrosc.* **2016**, *153*, 530–534, doi:10.1016/j.saa.2015.09.009.
6. Yang, L.; Li, M.; Wang, Y.; Zhang, Y.; Liu, Z.; Ruan, S.; Wang, Z.; Wang, S. An Isocamphanyl-Based Fluorescent “Turn-on” Probe for Highly Sensitive and Selective Detection of Ga^{3+} and Application in Vivo and in Vitro. *Analyst* **2021**, *146*, 7294–7305, doi:10.1039/D1AN01368H.
